# Constructing personalized longitudinal holo’omes of colon cancer-prone humans and their modeling in flies and mice

**DOI:** 10.18632/oncotarget.6463

**Published:** 2015-12-04

**Authors:** Myrofora Panagi, Konstantina Georgila, Aristides G. Eliopoulos, Yiorgos Apidianakis

**Affiliations:** ^1^ Department of Biological Sciences, University of Cyprus, Aglatzia, Nicosia, Cyprus; ^2^ Laboratory of Molecular and Cellular Biology, Division of Basic Sciences, University of Crete Medical School, Heraklion, Crete, Greece; ^3^ Institute of Molecular Biology and Biotechnology, Forth, Heraklion, Crete, Greece

**Keywords:** Drosophila, inflammation, cancer, microbiota

## Abstract

Specific host genes and intestinal microbes, dysbiosis, aberrant immune responses and lifestyle may contribute to intestinal inflammation and cancer, but each of these parameters does not suffice to explain why sporadic colon cancer develops at an old age and only in some of the people with the same profile. To improve our understanding, longitudinal multi-omic and personalized studies will help to pinpoint combinations of host genetic, epigenetic, microbiota and lifestyle-shaped factors, such as blood factors and metabolites that change as we age. The intestinal holo’ome – defined as the combination of host and microbiota genomes, transcriptomes, proteomes, and metabolomes – may be imbalanced and shift to disease when the wrong host gene expression profile meets the wrong microbiota composition. These imbalances can be triggered by the dietary- or lifestyle-shaped intestinal environment. Accordingly, personalized human intestinal holo’omes will differ significantly among individuals and between two critical points in time: long before and upon the onset of disease. Detrimental combinations of factors could therefore be pinpointed computationally and validated using animal models, such as mice and flies. Finally, treatment strategies that break these harmful combinations could be tested in clinical trials. Herein we provide an overview of the literature and a roadmap to this end.

## INTRODUCTION

Among cancers that affect both men and women, colorectal cancer (CRC) is the second leading cause of death in the United States and Europe. Interestingly, more than 90% of CRC cases occur in people 50 years or older. This fact is in line with the notion that sporadic cancers are diseases of old age and indicates that changes that accompany aging exert major influences on the biology and evolution of cancer. Nevertheless, the factors that change with age are not well understood. Mutations in *K-Ras, APC, p53* and other genes are well-known CRC-contributing factors and accumulate in tumors over time. However, these mutations accumulate at different rates in individuals and do not necessarily exert the same effects. One could therefore reason that additional, non-genetic risk factors may act in concert with genetic changes to drive sporadic CRC as we age.

Lifestyle is another factor contributing to CRC. The intestinal biochemical environment is shaped most prominently by dietary habits and by additional lifestyle factors [1, 2], including cigarette smoking [3], heavy use of alcohol [4], infections [5], stress [6], obesity [7] and physical inactivity [1]. These factors may induce detrimental genetic or epigenetic alterations and changes in the microbiota. Interestingly, adopting healthy lifestyle habits at an old age, including following CRC diagnosis, improves survival prospects, indicating that prior detrimental alterations can be counteracted [8].

Similarly, various intestinal microbes have been suspected to contribute to CRC by impacting enterocyte proliferation and death, modifying host metabolism, or by disrupting immunological homeostasis. However, assigning a role for any of them as a causative agent of CRC is complicated. For example, establishing a causative relationship between *Helicobacter pylori* and gastric ulcers causing gastritis and cancer needed to satisfy most of Koch’s postulates, i.e. be found and isolated from ulcers, proven to cause disease when introduced to a healthy organism (Barry Marshall, the Nobel laureate himself), and tackled through antibiotic treatment for ulcer eradication. It is even more difficult to establish Koch’s postulates with a complex microbial community, especially if some microbes cannot be readily cultured. 

Chronic inflammatory pathologies such as inflammatory bowel disease (IBD) provide examples of how genetic and nongenetic factors intersect to orchestrate disease pathogenesis. Accumulating evidence highlights the impact of an exaggerated immune response to intestinal microbiota and dysbiosis, or aberrant microbial community composition, in the development of IBD and potentially cancer [9]. The systemic inflammatory reactions to dysbiosis coupled with metabolic products of pathogenic bacteria establish a microenvironment rich in free radicals, DNA-damaging toxins, cytokines and growth factors that, collectively, foster tumor development [10]. While IBD preexists in only a small number of people with CRC, the role of inflammation in cancer might be broader than previously thought. A subclinical form of inflammatory signaling that contributes to heightened epithelial regeneration, as pointed by studies in flies and mice, may instead contribute to many of the CRC cases [11-13]. 

**Figure 1:**
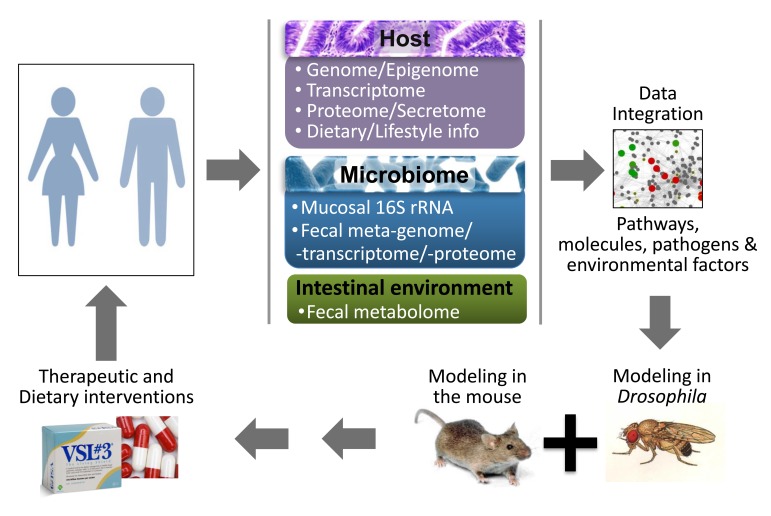
A roadmap to identify detrimental synergisms within human holo’omes as causal for colon cancer and develop personalized therapeutic or preventive strategies. A systems biology approach to assess shifts in intestinal holo’omes in humans and its link to colorectal pathologies will necessitate analysis of host intestine and microbial community genome, transcriptome, proteome metabolome and blood secretome. Using computational platforms, the genetic, metabolic, nutritional, microbial and immunological information accumulated, together with publicly available phenotypic and molecular function data, will be explored to obtain a ‘holistic’ view of key pathogenic processes and their hierarchies, to simulate the expected response to hypothetical interventions and develop new basic and translational research hypotheses. Reductionist approaches in *Drosophila* and mice - which can be genetically manipulated to express or lose the expression of specific genes in the intestine, while fed or injected with specific microbes and metabolites - could be used to assess detrimental synergisms of the intestinal holo’ome in driving inflammation and tumorigenesis, and guide the development of intervention strategies. Such therapeutic or dietary interventions could be translated to the clinic aiming to treat patients against microbial and intestinal environment imbalances as a means to alleviate intestinal inflammation and CRC.

**Figure 2:**
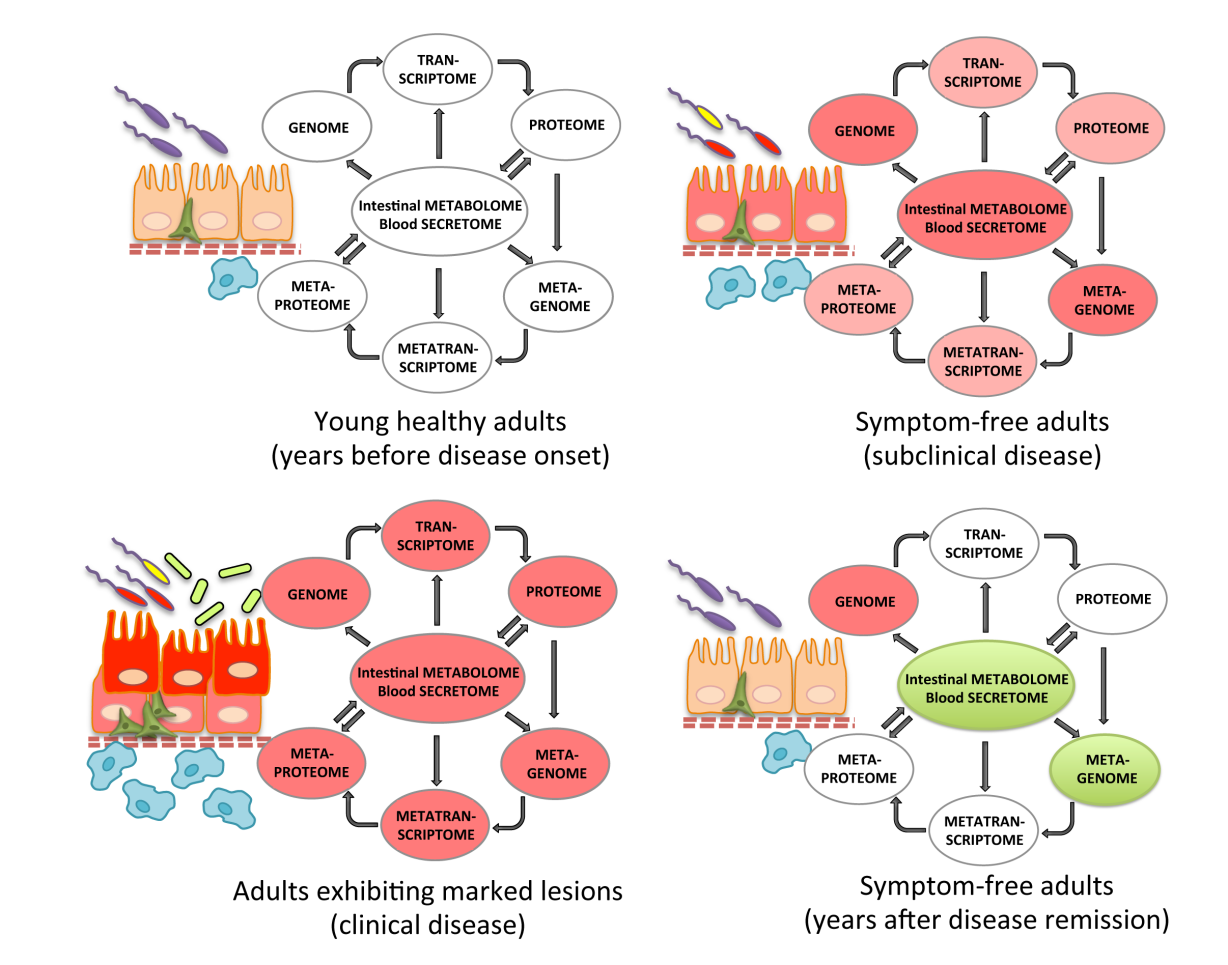
Recording the holo’ome longitudinally in humans. The holo’ome can be recorded via genomics, transcriptomics and proteomics of the host and the microbiota, metabolomics and proteomics of stool samples and secreted factors (e.g. cytokines and metabolites) of blood samples. To identify detrimental synergisms within the holo’ome, its parts need to be recorded around two points in time for each human individual: a. at a disease-free state, years before the onset of subclinical disease or long after disease remission, and b. upon the onset of subclinical disease. Disease remission may be facilitated by diet- or microbiota-based treatments that change the intestinal or blood environment or the microbiota.

The complex nature of CRC integrating genetic, epigenetic, environmental and microbial cues underscores the need for a holistic perspective and suggests that assessing these factors combinatorially on a personalized basis may be the key to pinpoint them. Moreover, CRC studies necessitate the use of simple model hosts that can reduce the complexity of the disease while reflecting key aspects of the human histopathology and concomitant molecular signals [14]. Mice and fruit flies possess these two key properties and are thus widely used. Based on data from human, mouse and *Drosophila* studies, the present review points to the importance of interactions among host gene expression, the intestinal microbiome and environment and systemic factors and metabolites, which comprise the intestinal holo’ome, an integral system controlling homeostasis, inflammation and cancer. As a roadmap for future studies on intestinal holo’omes we propose: a) a synthesis of information on individual human genome, transcriptome and proteome, the microbiota metagenome and metatranscriptome, the fecal metabolome and proteome and the blood secretome at critical time points, long before and upon the development of pre-cancerous lesions; b) the identification of the co-existence of factors as potential detrimental synergisms within holo’omes linked to disease onset; c) the validation of such synergisms using model organisms, such as flies and mice; and d) the assessment of therapeutics against such detrimental synergisms in clinical trials (Figures 1 and 2).

## THE INTESTINAL HOLO’OME

### The host genome and epigenome in intestinal inflammation and cancer

An early step in CRC is the development of polyps. Polyps, the aberrant growth of cells within the colorectal epithelial mucosa, can be benign (non-dysplastic) or dysplastic. If dysplastic, these are referred to as premalignant adenomas. Adenomas proceed to malignancy when they invade the underlying tissues (lamina propria) and successfully form secondary tumors to distant sites (metastasis). In addition, genomic instability contributes to tumorigenesis due to defects in DNA repair systems and the concomitant increase in the rate of mutations [15, 16]. The transition from a normal to hyperplastic epithelium is frequently linked to the inactivation of the *adenomatous polyposis coli (APC)* tumor suppressor. *K-Ras* or *B-Raf *oncogene activation leads to the formation of large adenomas [17]. Late adenomas may associate with loss of *SMAD4, *a key component of the transforming growth factor beta (TGFβ) signaling pathway, which normally suppresses tumor growth [18, 19]. The progression from large adenomas to cancer may also require a mutation in the *p53 *locus [20]. Loss of function of the tumor suppressor *PTEN *and subsequent upregulation of the PI3K/AKT signaling pathways, also facilitate the development of colon cancer [21]. Tumor metastasis can be further facilitated by PRL3 overexpression, a gene involved in malignant tumor cell motility and metastasis [22]. All the aforementioned genes have homologues in *Drosophila* and similarly to mammals, *Drosophila Apc* loss of function and *K-Ras/Ras1* oncogene promote disease and may synergize during intestinal tumorigenesis [23]. Interestingly, many *Drosophila* studies point to a highly conserved JNK-Hippo-JAK/STAT pathway axis that promotes tumorigenesis in many cases, for example, upon synergism between the *Ras1* oncogene and cell polarity gene mutants [24, 25]. Such a pathway axis is yet to be established in mammals.

In addition, there is a strong genetic basis for mutations and polymorphisms linked to increased inflammation in the intestine of flies, mice, and humans. For example, frame-shift mutations within the *NOD2* locus may cause IBD *via* impaired NF-κB activation in response to bacterial peptidoglycan [26]. Similarly, the Asp299Gly polymorphism in the TLR4 is associated with IBD, due to impaired NF-κB activation by Gram-negative bacteria [27]. Mutations of the autophagy gene Atg16L1 in combination with viral infection induces intestinal pathologies in mice resembling those observed in IBD patients [28]. Polymorphisms within the pro-inflammatory cytokines IL-1, IL-6 and IL-22 and STAT3 pathway activation may boost mucosal cytokine expression and enterocyte regeneration, thereby facilitating gastrointestinal cancer [29-31]. Strikingly, STAT, Imd/NF-κB, autophagy and NADPH oxidase pathways play a pivotal role in both mammalian and *Drosophila* innate immunity and intestinal host defense [11, 32-35]. 

Gender itself is a genetic variation that affects inflammation and cancer in the intestine. At all ages, women are less likely than men to develop sporadic colon cancer [36], an observation that has been recapitulated in the *Apc*^Min/+^ CRC mouse model and attributed to tumor-promoting effects of testosterone [37]. By contrast, estrogens dampen inflammation and protect from colitis and colitis-associated cancer in mice [38, 39], in line with the reduced severity of IBD in postmenopausal women receiving estrogen hormone replacement therapy [40]. 

In addition to genetic alterations, the transition from normal mucosa to adenomatous polyps is marked by epigenetic changes, namely DNA methylation, histone modifications and aberrant expression of non-coding RNAs^17^. These epigenetic events include hypermethylation and silencing of a number of genes with a proven contribution to CRC including genes of the Wnt signaling pathway such as *APC*, *WNT5A* and *AXIN2* and the DNA repair genes *MLH1*, *MLH2* and *MGMT* among others [41]. Importantly, high-throughput methylation profiling has indicated the existence of three epigenetic subtypes characterized by high, intermediate, and low methylation that exhibit particular clinicopathological and molecular features [41]. Thus, CpG island methylator phenotype 1 (CIMP1) tumors that are typified by high DNA methylation levels are associated with microsatellite instability (MSI) and *B-Raf* mutations. CIMP2 tumors show frequent *K-Ras* but not *B-Raf* mutations or MSI and CIMP-low/negative CRC display high frequency of p53 mutations. A recent study by Akhtar-Zaidi *et al.* has also implicated histone modifications in CRC by identifying changes in Lys4-methylated histone 3 (H3K4me1) as drivers of a transcriptional program that promotes carcinogenesis in the colon [42]. Intestinal inflammation may significantly contribute to epigenetic reprogramming affecting all stages of CRC. For example, IL-6 regulates the expression and activity of DNA methyltransferase 1 (DNMT1) leading to enhanced methylation of tumor suppressor genes [43, 44]. IL-6 also engages a STAT3 pathway that suppresses the expression of miR-34a releasing its inhibitory control over the IL-6 receptor. This epigenetic switch results in amplification of IL-6 signaling and the establishment of a feedback loop that promotes EMT, invasion and metastasis [45]. 

Links between the genome and epigenome in orchestrating intestinal pathologies are also beginning to emerge. Of note, an IBD susceptibility SNP variant of IL-23 receptor (IL-23R) exhibits reduced ability to bind microRNAs Let-73 and Let-7f, leading to aberrant IL-23R expression and deregulated signaling relevant to IBD pathogenesis [46]. However, the type of epigenetic modifications, the timing and causality to CRC and IBD remain poorly defined and utilization of simple models amenable to genetic manipulation such as *Drosophila* are warranted to define how genomic and epigenetic events intertwine to control intestinal pathologies. 

### The intestinal microbiome

The mucosal epithelium is in continuous contact with a myriad of autochthonous (resident) and allochthonous (transient in the fecal stream) microbes. In humans, the density of microbes is approximately 10^12^ bacteria per gram of dried colonic content [47]. Microbial colonization begins immediately after birth and the composition changes, over the first two years, to reach a steady community whose composition is defined by many factors, including immune responses, enterocyte turnover, intestinal motility, pH, redox status and nutrient availability [48]. For instance, during the neonatal mammalian life, the intestinal environment is characterized by a reduced oxidation potential that favors the growth of facultative anaerobes, such as streptococci and coliforms [49]. Following weaning, the microbial community becomes more dense and diverse as the high-fat milk diet is replaced by a high-carbohydrate diet. In mice, the mature microbiome is mainly defined by Firmicutes, Bacteroides and Proteobacteria [48]. The mucosal layer of the mouse large intestine is highly enriched for the phylum Firmicutes and, more specifically, for the families Lachnospiraceae and Ruminococcaceae, whereas families such as Bacteroidaceae, Enterococcaceae and Lactobacillaceae are enriched in the mouse lumen [48]. Similarly, the human colon is dominated by four phyla, namely, Firmicutes, Bacteroides, Proteobacteria and Actinobacteria [50]. Gender associations of the gut microbiome composition in healthy humans remain inconclusive, likely reflecting strong environmental influences [51]. Of note, however, specific taxa of Actinobacteria, Proteobacteria, and Firmicutes express enzymes that have the capacity to metabolize steroid hormones and influence their activity [52, 53]. Whether microbiome-derived sex steroids impact on host immunity in a manner similar to that of host-derived hormones and, indirectly, through changes in intestinal microbiome composition remains unknown. Interestingly, a bi-directional association between testosterone levels and microbial communities in the mouse gut has been noted and linked to protection from Type 1 diabetes [54]. 

The *Drosophila *is most frequently colonized by Lactobacillales and Acetobacteraceae and occasionally by Enterobacteriaceae, which belong to the Firmicutes and Proteobacteria phyla, but it lacks Bacteroides and other obligate anaerobes presumably due to the presence of oxygen in the fly gut [55]. However, there is significant bacterial variation in terms of diversity and density along the gastrointestinal tract in mammals and in flies [56]. Bacteria populations are more dense in the small and large intestine than in the stomach [48] partly because ingested bacteria die in the acidic environment of the stomach. Thus, areas with approximately neutral pH in the mammalian small and large intestine or the anterior and posterior *Drosophila* midgut might offer a more conducive environment for colonization [48, 56]. Despite the similarities at the level of Firmicutes and Proteobacteria phyla between humans and mice or invertebrates, there are profound differences even among or within individuals of the same species, longitudinally over time and upon various treatments or diets as one moves towards the bacterial species level [50, 57]. This variation makes analysis of human microbiota very complicated; therefore, simple model organisms can be useful in elucidating the contributions of microbiota in inflammation and cancer. For example, NF-κB signaling in the *Drosophila* intestine directly decreases the abundance and modifies the structure of microbiota, while NAPDH oxidase/Duox signaling decreases microbiota abundance and causes oxidative stress to the midgut epithelium [58].

#### Benefits and problems of having intestinal microbiota

In the absence of intestinal immunological imbalances or pathogenic microbiota, intestinal microbes are largely considered beneficial or neutral. These bacteria are in constant competition for intestinal niches, which is very important for fending off *bona fide* or opportunistic enteric pathogens and operate synergistically in order to maintain the overall community function [59]. Furthermore, the metabolic by-products of one species may support the growth of other species or inhibit the colonization by other potentially harmful microbes [50, 60]. Bacteria can also affect the host metabolism while benefiting from the nutrient-rich niche of the intestine. For example, humans lack cellulases and therefore need intestinal bacteria to digest plant cellulose. The mammalian host also takes advantage of the terminal products of microbial fermentation, such as butyrate, acetate and propionate as energy sources [61, 62]. In addition, these bacterial short-chain fatty acids (SCFAs) can act as immunomodulators that contribute to immune homeostasis while suppressing the secretion of pro-inflammatory cytokines [62]. Similarly, in *Drosophila*, *Lactobacillus plantarum* and *Acetobacter pomorum* have been shown to contribute to the nutrition of the host upon nutrient-poor diet. For example, colonization of axenically reared embryos with *L. plantarum *promotes growth when nutrients are limiting by activating the TOR signaling pathway which improves viability and accelerates the developmental rate [63]. Similarly, *A. pomorum *enhances growth of larvae under nutrient scarcity *via* the alcohol dehydrogenase PQQ-ADH, which is required for acetic acid production by the bacteria and the subsequent host insulin pathway activation [64]. On the other hand, *Drosophila* infection with *Vibrio cholerae* leads to inactivation of insulin/insulin-like growth factor signaling (IIS) and lipid accumulation in enterocytes *via* intestinal acetate depletion [65].

Intestinal microbiota may also contribute to the maintenance of mucosal barrier integrity. For example, symbiotic bacteria are capable of suppressing the activation of NF-κB pathway in intestinal epithelial cells by inhibiting the ubiquitination of IκB, the inhibitory molecule of NF-κB. Additionally, they may block NF-κB signaling by facilitating the nuclear export of NF-κB subunit, p65, *via* regulation of the peroxisome proliferator-activated receptor (PPAR)γ [66]. Considering the contribution of resident bacteria in host defense mechanisms, polysaccharide antigens produced by *B. fragilis* promote CD4^+^ T cell expansion and cytokine production [67]. Furthermore, *Lactobacillus spp.* modulate the activation of dendritic and natural killer cells [68, 69]. Strikingly, in both flies and mice *Lactobacillus spp.* are important in maintaining a baseline of intestinal regeneration of the intestine as a mechanism of host defense *via* the induction of reactive oxygen species [33, 70]. 

The beneficial role of microbiota is clearly demonstrated in germ-free animal models. Animals raised in germ-free conditions have acute developmental and immunologic deficiencies e.g. altered intestinal morphology defined by a reduced muscle wall thickness and underdeveloped villus capillaries [71]. The decrease in angiogenesis is attributed to the limited expression of Angiogenin-4, a potent stimulator of new blood vessels [72] which can be specifically induced by* Bacteroides thetaiotaomicron *[73]. Host-specific commensal bacteria are required for the expansion of T cells and consequently, for the full maturation of intestinal immune system. For example, segmented filamentous bacteria are involved in intestinal T cell expansion [74], IgA activation and induction of epithelial MHC-II expression [75]. Interestingly, the differences between mouse and human microbiota appear to be functionally important because, for instance, colonization of mice with human microbiota results in an immature innate and adaptive immunity and greater susceptibility to infection, as seen in germ-free mice [74]. Germ-free animals are also characterized by impaired cytokine and antimicrobial peptide production, smaller Peyer’s patches, fewer intraepithelial lymphocytes and IgA secretion and thus, they are more vulnerable to infections [76]. Germ-free mice show aberrant nutrient absorption presumably due to a decreased metabolic rate and limited digestive enzyme activity, and as a result they tend to consume more calories to maintain a normal body weight [77]. Interestingly, re-colonization of germ-free animals with an intestinal microflora is sufficient to restore many of those functions [78]. Similarly, mono-colonization of germ-free animals with the human commensal bacterium *Bacteroides fragilis* suffices to restore the CD4^+^ T-cell development through the expression of the microbial molecule polysaccharide A (PSA) [79] . Moreover, inoculation with the single gut inhabitant *Bacteroides thetaiotaomicron *[71] or *B. fragilis *[79]**can stimulate villus capillary formation and promote intestinal development. Therefore, the presence of the “right microbes” in the “right host and environment” may determine gut homeostasis.

Various conditions may lead to intestinal dysbiosis, a change in the microbiota composition that is unfavorable for the host and/or immunological imbalances, such as an exaggerated chronic response to the microbiota. Dysbiosis has been reported in a number of enteric disorders and great efforts have been made to define the microbial communities in the intestine of diseased individuals. Bacterial 16S ribosomal RNA and whole genome sequencing studies have linked numerous yet uncultured microorganisms to intestinal disease [80, 81]. Certain bacterial species are prevalent among colon cancer patients. These autochthonous bacteria include *Streptococcus gallolyticus *[82], enterotoxigenic* Bacteroides fragilis *[83],* Escherichia coli *[84] and *Fusobacterium nucleatum *[85]. Similarly, the relative abundance of *Proteobacteria*, such as *E. coli* and other* Enterobacteriaceae*, compared to other phyla is linked to IBD [86]. However, it is difficult to determine whether these alterations refer to the pre- or post-disease state. Alterations within the intestinal ecosystem secondary to pathogen invasion, chronic inflammation or antibiotic treatment may influence the availability of nutrients in the intestinal environment, deregulate the immune response and promote the colonization of opportunistic pathogens [87]. 

IBD studies in model systems demonstrate that the breakdown of immune tolerance towards indigenous bacteria could lead to inflammatory colitis. For example, immunocompromised mice deficient for T-bet, a transcription factor that orchestrates inflammatory genetic programs in both adaptive and innate immunity, develop IBD that largely resembles human ulcerative colitis [88]. Surprisingly, this colitic phenotype could be transmitted not only to the progeny but also to unrelated wild-type animals, indicating that the presence of an aberrant microbiota is sufficient to cause colitis. Similarly, deregulation of innate immunity against intestinal microbiota in flies *via* mutations of negative regulators of intestinal innate immune response or *via* senescence-related deregulation of innate immunity leads to intestinal dysbiosis and intestinal dysplasia-like phenotypes [60, 89]. 

Changes in microbiota composition have also been documented during colorectal carcinogenesis. In an established mouse model of colitis-propelled CRC induced by the combined application of the mutagen azoxymethane (AOM) and the luminal toxin dextran sodium sulfate (DSS), the progression from chronic inflammation to dysplasia and adenocarcinoma was associated with significant shifts in microbial community structure, for example of *Prevotella*, *Porphyromonadaceae* and *Bacteroides* genera [90]. Notably, the late stages of colitis-associated CRC in this model were typified by enriched populations of *Erysipelotrichaceae* of the phyllum Firmicutes and colonization of germ-free mice with tumor-associated gut microbiome exacerbated tumorigenesis in these animals [90]. In another model of CRC induced by application of AOM to colitis-susceptible *Il10*^−/−^ mice lacking the immunoregulatory cytokine IL10, commensal polyketide synthase (*pks*)-positive *E. coli* were found to accelerate progression from dysplasia to invasive carcinoma [91]. Whilst the role, if any, of microbiota perturbations in metastasis remains poorly studied, the abundance of *Fusobacterium nucleatum* in human colon tumors has been reported to associate with lymph node metastasis [85]. Together, these observations imply that changes in microbiota composition may impact on different stages of colorectal carcinogenesis. However, the identification of autochthonous bacterial members as pathobionts (inflammation-/tumor-promoting) or beneficial (inflammation-/tumor-suppressing) would require longitudinal studies in human individuals i.e. long before and upon the onset of disease and monitoring during disease progression [92, 93].

### Environmental factors affecting intestinal dysbiosis

The intestinal biochemical environment plays a fundamental role in sustaining a “healthy” host-microbiota interplay. Approximately 5% of people in the United States will develop CRC and half of them will die from the disease [94]. About 75% of the diagnosed CRC cases are sporadic, that is, not evidently hereditary. Thus, beyond genetics, the environment plays a critical role in cancer [2]. Western pattern diet and lifestyle, heavily processed food, frequent use of antibiotics and apparently the improved hygiene in industrialized countries are among the key environmental factors that adversely affect microbiota composition and its interaction with the host. 

#### Diet

Diet is a source of both gut-colonizing bacteria and nutrients that can rapidly alter microbiome structure [95]. The impact of diet has been studied in mice after they have switched from a diet low in fat and high in complex polysaccharides to a westernized diet, rich in fat and sugars. Within a day, the mice display distinct alterations in microbiota composition, gene expression and metabolic pathways, and develop significant adiposity within two weeks [96]. Moreover, diets limited in simple sugars enable mouse intestinal microbiota to outcompete pathogenic *Citrobacter rodentium* [97]. Similarly, the *Drosophila* intestinal microbiota interacts with dietary ingredients to produce vitamin B and proteins or modify the lipid/carbohydrate storage of the host [98]. Interestingly, fly studies show that diet preference and bacterial intestinal colonization level can be affected by the presence of bacterial metabolites in the fly food [99]. Such feeding behaviors and metabolites may have a profound influence on the establishment of intestinal microbiota and the shape of the intestinal biochemical environment. Moreover gender-specific effects of diet on gut microbiota composition and metabolism have been reported across different vertebrate species. For example,* Lactobacillus* and *Clostridium* are more abundant in male mice fed a high-fat rather than chow diet, whereas in females these genera are less abundant in high-fat diets [100]. In *Drosophila, *aspects of the interaction between the microbiota and the host metabolic programs, such as energy storage and protein content, are also sex specific [98]. 

Changes in diet that modify microbiota may affect the development of inflammatory and malignant diseases. For example, high fat diet promotes the expansion of intestinal bacterium *Bilophila wadsworthia *and colitis in IL10-deficient mice [101]. In addition, lower dietary fiber intake precedes the development of inflammatory pathologies by reducing the production of microbial immunomodulatory products, such as SCFAs [102]. SCFAs selectively expand IL10-producing regulatory T cells (T_reg_) in the intestine, which in turn suppress inflammation [103]. Interestingly, the SCFA effects on T_reg_ are mediated in part by histone acetylation of the *FoxP3* (forkhead box P3) locus leading to elevated expression of FoxP3, a transcription factor required for the differentiation of CD4^+^ T lymphocytes to T_reg _[104, 105]. These findings suggest that microbial metabolic products of diet epigenetically modulate host gene expression and hint to important links between commensal microbiota and epigenetic changes in the immune system that may influence the onset of inflammation and cancer in the intestine. In line with this hypothesis, high levels of *Fusobacterium* that typify both IBD and CRC, correlate with aberrant CpG island methylation in inflamed and malignant tissue [106, 107]. However, as *Fusobacterium* is also part of the normal microbial ecosystem and is not associated with DNA methylation in cancer-free subjects, further studies are required to establish causative links between *Fusobacterium* species and epigenetic re-programming of the host and to identify putative co-factors that enable them to promote intestinal disease.

In terms of carcinogenesis, a number of studies have demonstrated a correlation between saturated fats and CRC [108, 109]. Dietary fat intake increases the production of bile acids. The primary products of bile acid metabolism are synthesized in the liver, where they get conjugated with glycine and taurine. These products get deconjugated by colonic bacteria to form secondary bile acids, namely lithocholic and deoxycholic acid. Accumulating evidence indicates that patients with CRC have elevated amounts of fecal lithocholic and deoxycholic acids compared to healthy controls [110]. Lithocholic and deoxycholic acids stimulate the production of reactive oxygen and nitrogen species, and the activation of the NF-κΒ signalling pathway [111, 112]. Chronic exposure to these secondary bile acids may enhance mutagenesis and increase epithelial cell proliferation and/or survival. Taken together, lithocholic and deoxycholic acids could be considered as proinflammatory and procarcinogenic bacterial metabolites. Further understanding of the interactions between indigenous microbiota and intestinal metabolites could thus take us one step forward in elucidating the complex relationship among diet, microbiota and colorectal pathologies. 

Accumulating evidence indicates that probiotics could be used as therapeutic strategies for the treatment of metabolic syndromes and chronic inflammatory diseases associated with aberrant gut microbiota [113]. Manipulation of microbiota through probiotic intervention may enhance resistance to intestinal colonization by pathogenic microbes, improve intestinal barrier function, increase the metabolism of nutrients and modulate immune responses [114]. For instance, the lactic acid bacteria *Lactobacillus plantarum *and* Lactobacillus brevis* may inhibit the secretion of pro-inflammatory cytokines and degrade bacterial glycosaminoglycan in a chemically induced colitis mouse model [115]. Inoculation of mice with *Lactobacillus acidophilus *early in life enhances host defense and prevents *Citrobacter rodentium* induced colitis [116]. Despite the lack of adaptive immune responses that may mediate the beneficial responses to *Lactobacillus* species, fly autochthonous bacteria, such as *L. plantarum,* but also pathogens, such as *Pseudomonas aeruginosa*, induce regenerative inflammatory signaling *via* the highly conserved JNK and STAT pathways, as part of the host defense response against intestinal infection [11, 117-119].

Probiotics have also been tested for their ability to prevent intestinal carcinogenesis in mouse models, with promising results [120]. Beyond the role of dysbiosis in the development of CRC, the gut microbiota impacts the therapeutic activity of anticancer agents by influencing pharmacodynamic and immunological parameters that define drug bioavailability and shape the tumor microenvironment respectively [121]. Manipulating the composition of gut microbiota through probiotics, prebiotics (that is, non-digestible agents that stimulate the growth and/or functions of specific microbiota components) and other dietary interventions may thus hold promise for the improved management of cancer patients. A better characterization of the interactions between bacterial species using axenic and gnotobiotic *Drosophila* and mouse models will facilitate this goal. 

Differences in the gut microbiota are more striking between different geographic areas, presumably because they encompass both genetic e.g. race and dietary differences. For instance, the microbiota of some African children fed with a diet rich in fiber, as compared to some European children, were found to be enriched in *Bacteroidetes, Xylanibacter* and *Prevotella *species and poor in *Enterobacteriaceae *[122]. The same African children produced significantly more SCFAs in their intestinal lumen [122]. Japanese, on the other hand, despite high standards of hygiene, do not show high allergy incidence (as the hygiene hypothesis would dictate), presumably due to the high intestinal levels of SCFAs, which are produced by the gut microbiota as a byproduct of fermentation of dietary fiber [102]. Nevertheless, other environmental factors, such as differences in pharmaceutical treatments may contribute to the microbiota composition [96]. 

#### Antibiotics and other drugs

Antibiotic treatment affects the gut microbiota abundance and diversity at the level of bacterial species. Bacterial communities more vulnerable to common antibiotics are reduced or lost allowing other communities to expand. As a result, intestinal dysbiosis may develop by the expansion of opportunistic pathogens. For example, antibiotics are usually associated with the expansion of *E. coli*, an inhabitant of the mammalian and invertebrate intestine [123]. An increase in the intestinal *E. coli *population is associated with the onset of IBD [124]. Drugs may assist pathogens indirectly by reducing the competitiveness of the host and the healthy microbiota against indigenous opportunistic pathogens. For instance, patients receiving chemotherapy or antibiotics are more vulnerable to *P. aeruginosa* infections, primarily due to compromised host immunity and altered intestinal microbiota [125, 126]. Similarly, antibiotic treatment in mice reduces the resistance of animals to intestinal colonization with *P. aeruginosa *[126]. Of note, endogenous *P. aeruginosa *can cross the intestinal barrier, translocate to and infect other organs, thereby causing systemic inflammation [127].

Although most of the bacterial families manage to re-colonize the gut following an antibiotic regime, the time in between is particularly critical for the host health, because antibiotic-resistant or -tolerant microbes may expand and become established for years [50, 128]. An example of this situation is the persistence of *Staphylococcus epidermidis* following clarithromycin treatment [129]. Since most antimicrobials cannot discriminate between pathogenic and non-pathogenic bacteria, dysbiosis is a likely result of extensive antimicrobial treatments. Similarly, frequent transfer of flies on new food containing preservatives eliminates most of their intestinal bacteria [130]. Thus identifying the microbial populations that become more abundant or virulent following antimicrobial treatments should be a serious consideration. 

#### Hygiene hypothesis

Strachan was the first to mention the role of hygiene in disease predisposition [131]. He coined the term “hygiene hypothesis” in his attempt to elucidate how the decreased exposure to infectious agents in early childhood, as a result of improved hygiene, could lead to an allergic incidence later in life. According to epidemiological studies, a dramatic increase in colon cancer, IBD, type 1 diabetes, atopy and asthma incidence has been observed during the past 50 years, principally in industrialized countries [132-134]. Communities with low socioeconomic status do not show a similar increase in disease incidence, implying that the immune system becomes educated by experiencing a range of microbes throughout life and consequently, acquires tolerance to relatively innocuous microorganisms [135]. Notwithstanding fundamental issues of hygiene, Tanzania’s hunter-gatherer Hadzabe people often consume the uncooked stomachs and colons of killed animals which may increase gut microbial diversity to the benefit of maintaining health in their ecosystem [136]. In developed countries vaccination and antimicrobial therapy must be taken into consideration to better understand and explain these population-based observations. Nevertheless, exposure to infectious agents early in life, may promote the development of regulatory T cells which in turn attenuates the inflammatory response *via* the induction of IL10 and transforming growth factor (TGF)-β1. For example, induction of IL10 following infection with enteric helminthes in mice protects against particular food allergens [137]. Further studies are warranted to address which microbes and anti-inflammatory responses are able to protect against chronic intestinal inflammation.

## ANIMAL MODELS IN INFLAMMATION AND CANCER

Various organisms may be used to model aspects of pathophysiology of human intestinal inflammation and cancer [138, 139]. Among them primarily rodents and secondarily flies are the most popular because they combine feasibility and significant similarity to humans. The mouse is highly conserved in many aspects of the human disease and well streamlined for various small-scale experiments. *Drosophila* on the other hand shares surprisingly high similarity to humans regarding disease related genes and signaling pathways while being less complex, which is an advantage for studying some of the basic principles of disease biology and performing large-scale *in vivo* studies on inflammation and cancer [11, 14, 140].

### Commonalities and differences between mouse and *Drosophila* intestinal anatomy and physiology

The mammalian and invertebrate gastrointestinal tracts are defined by unique compartments, each of which is responsible for the execution of distinct biological processes. The fly intestine is segregated into five main compartments; the foregut, the crop, the midgut, the malpighian tubules and the hindgut [141]. The foregut corresponds to the mammalian esophagus, whereas the crop and the acidic copper cell region in the middle of the midgut stores and helps digesting food, respectively, thus sharing similarities with the mammalian stomach. The midgut corresponds to the fast renewing mammalian small intestine where the majority of digestion and nutrient absorption takes place [142-144] and is further subdivided into various anterior and posterior regions of distinct expression profiles and stem cell regulation [145, 146]. The malpighian tubules have renal-like properties and are located at the midgut-hindgut boundary. Hindgut is the last compartment of invertebrate intestine and corresponds to the slow, damage-induced renewing property of the mammalian large intestine [144, 147]. The hindgut is the tissue where water and ions most likely get absorbed and the fecal content is promoted to the rectum for excretion [14, 148]. 

The *Drosophila *midgut is a linear tube that lacks the mammalian intestine crypts and villi. Nevertheless, both of these tissues are of endodermal origin, containing an epithelial monolayer of cells. Enterocytes (ECs) are the most abundant cells in the intestine and have absorptive functions, while secretory enteroendocrine cells (EE), intestinal stem cells (ISCs) and enteroblasts (EBs), account for the minor cell populations of the gut epithelium [143]. Similarly to the mammalian gut, ISCs are maintained by Wg/Wnt signaling and divide asymmetrically to give rise to transient cells, the EBs, and new ISCs or divide symmetrically to increase the number of ISCs [149, 150]. In *Drosophila*, transient cells do not undergo any cell division but they differentiate into either an EC or an EE, while mammalian transit amplifying cells also produce the goblet and Paneth cells, which secrete mucus and antimicrobial peptides, respectively [14, 144]. 

In both *Drosophila *and mammals*, *a layer of basement membrane underlines and supports the intestinal epithelium and an outer musculature confers intestinal motility. In mammals, however, three additional layers are found in sequence between the outer musculature and the basement membrane: the submucosa, the muscularis mucosae and the lamina propria. The latter contains immune cells and specialized immunity tissues, such as the Peyer’s patches [14]. Despite the lack of adaptive immunity as we know it in humans and mice, *Drosophila* phagocytes accumulate outside the adult midgut upon infection contributing to regenerative inflammatory signaling [12]. Moreover, the *Drosophila *tracheal system has been paralleled with the mammalian circulatory system, since both have been characterized as branched tubular networks that transport gasses to all organs, although the *Drosophila *tracheal system does not transport blood as in mammals [151]. In addition, the diversity of gut microbial community in flies is a hundred times lower than in mammals, and is totally devoid of obligate anaerobes [148]. Therefore, flies offer a basic but not the complete inflammation-tumor microenvironment as it occurs in humans. Accordingly, fly models can serve as a point to accelerate the discovery of basic principles that govern cancer, but the mouse is more suitable for the investigation of specialized aspects of the intestinal inflammation and cancer that depend, for example, on obligate anaerobes, the adaptive immunity, secretion of bile acids and specialized cells of submucosa or lamina propria. 

### Regenerative inflammatory signaling and tumor modeling in the *Drosophila* intestine reveals synergisms among the host, its microbes and the intestinal chemical environment

The contribution of *Drosophila* to cancer research is instrumental [152, 153]. Up to 75% of genes that associate with human diseases, including cancer, have functional homologues in *Drosophila *[154]. In addition, the *Drosophila* genome has fewer genes compared to the human genome and a lower genetic redundancy, making the identification of disease-related signaling pathways easier. Thus, *Drosophila* studies have identified many genes and signaling pathways before their human counterparts were linked to cancer. For example, Notch pathway mutant flies were first identified due to a phenotype of notched wings. Years after the initial characterization of the notched phenotype in flies, the human homologue Notch1 was found to cause T cell lymphoplastic leukaemia [155]. Likewise, the hedgehog and hippo signaling pathways, which play a role in human tumorigenesis, have been initially studied in *Drosophila *[156]. Pertinent to human leukemia and CRC, fly studies were the first to demonstrate the role of constitutive JAK-STAT signaling pathway activation in hematopoietic disorders and intestinal regeneration [119, 157]. 

Due to the great availability of genetic tools that enable the modulation of gene expression in a time and tissue specific manner [140], tumor modeling in *Drosophila *is relatively easy and robust. Tumors can be easily induced in larvae and adult flies following constitutive or conditional knockout of tumor suppressor genes, such as cell polarity growth control regulators. For example, loss of *scribbled* and *salvador* tumor suppressors cause the transformation of renal stem cells into dysplastic-like tumors in the adult *Drosophila *malpighian tubules [158]. Carcinogenesis in flies can be modeled also by using gain of function conditions similar to those leading to tumor development in humans [153]. For instance, cancer models combining oncogenic Ras activity and mitochondrial dysfunction lead cooperatively to excessive ROS generation. This in turn activates a Wnt/Wg and JNK signaling pathway which inactivates Hippo and upregulates the IL6-like Upd leading to tumorigenesis [159].

*Drosophila *studies have also advanced our knowledge on intestinal response to infection and damage and the concomitant intestinal regenerative inflammatory signaling [11, 151]. Under pathogenic conditions, such as EC damage and stress or aging, a series of highly conserved *Drosophila *signaling pathways, including the EGFR, Wnt/Wg, PDGF/PVF2, INSR/InR and the JNK-Hippo-JAK/STAT, induce stem-cell driven regeneration [117, 160-166]. Regeneration necessitates ISC proliferation and differentiation, as a compensatory defense response replenishing damaged cells [70, 117]. Nonetheless, perturbations of this response, due to mutations, aging or imbalances within the microbiota may lead to an overproduction of differentiating cells and tissue dysplasia-like phenotypes [14, 60, 117, 118, 161]. While these phenotypes that accrue during aging are reminiscent of spontaneous tumors [167], they are not invasive and it remains to be established if they are a result of spontaneous mutations as in humans. 

Moreover, intestinal infection with *P. aeruginosa *in* Drosophila* activates the c-Jun N-terminal kinase (JNK) pathway, which causes apoptosis of enterocytes and leads to proliferation of ISCs [117]. Strikingly, genetic predisposition *via* a K-Ras/Ras1 oncogene expression in ISCs synergizes with *P. aeruginosa-*induced inflammatory signals promoting stem cell-mediated tumorigenesis. Interestingly, *P. aeruginosa* virulence against *Drosophila* is enhanced when exposed to peptidoglycan derived from human commensal Gram(+) bacteria [168]. Moreover, sustained intestinal infection with *P. aeruginosa* in *Drosophila* induces the NF-κB/Imd pathway, which synergizes with the Ras1^V12^ oncogene to activate the JNK pathway. This synergism leads to invasion and dissemination of oncogenic hindgut cells to distant sites [169, 170]. Another striking example of microbe-gene synergism is the overabundance of the intestinal pathobiont *Gluconobacter morbifer* upon persistent NF-κB/Imd pathway activation, which in turn induces the NADPH oxidase Duox to produce reactive oxygen species and concomitant hyperplasia [58].

Additional studies reveal a role for *Drosophila* phagocytes in solid tumor biology. *Drosophila* phagocytes, named plasmatocytes, are responsible for the engulfment of apoptotic cells and invading pathogens.**Tumor models are used for studying the role of these cells in tumor progression [171]. For example, double mutant *Ras^V12^/scrib^−/−^* tumors within the *Drosophila* larval tissues increase the number of circulating plasmatocytes and attract them to the tumor site. Invasive tumors and concomitant tissue damage activates JNK signaling, which in turn induces JAK/STAT pathway-activating cytokines. These cytokines are amplified by additional cytokine expression in circulating plasmatocytes and the fat body [172]. Moreover, expression of the cytokine TNF by circulating plasmatocytes stimulates the JNK pathway and subsequently matrix metalloproteases in malignant cells, which assist tumor invasiveness [173]. While these studies do not address the role of plasmatocytes in the intestine, *Drosophila* phagocytes were recently shown to accumulate in the *Drosophila* midgut upon infection or oxidative stress contributing to regenerative inflammatory signaling [12].

### Inflammation and tumor modeling in the mouse intestine reveals synergisms among the host, its microbes and the intestinal environment

Historically, the contribution of rodent models in cancer research begun with the generation and maintenance of mouse strains, inbred to the extent of total homozygosity. Spontaneous mutations arising within these inbred strains provided fundamental information regarding basic mechanisms of carcinogenesis. The laboratory mouse *Mus musculus* is the most frequently used animal model in *in vivo *cancer studies primarily because approximately 99% of human genes have murine orthologues [174]. Even more frequently than in flies, human disease genes display an analogous role in mice [175]. Despite the increased complexity as compared to flies, the mouse genome can be manipulated experimentally and carcinogenic, microbial and inflammatory agents can be combined to study inflammation and cancer in the intestine [176]. Available models integrating dysbiosis, inflammation and tumorigenesis broadly fall into 2 groups: (a) Models in which epithelial integrity disruption is the primary event, for example, following administration of the luminal toxin dextran sodium sulfate (DSS), or by genetic ablation of the NF-κB regulatory gene *IKK*γ*/NEMO*, or by loss of heterozygosity of the *APC* tumor suppressor gene in intestinal epithelial cells. The ensuing tissue damage allows the translocation of bacteria from the lumen into the mucosa triggering a potent colitis-like inflammatory response or in the case of APC, aberrant cell proliferation. (b) Models in which inflammation is the primary pathological event due to either genetic disruption of immunological balance (e.g. IL10 or combined T-bet/Rag2 deficiency) or introduction of pathogenic bacteria (e.g. *Helicobacter hepaticus*causing dysbiosis. Damage to the epithelium is likely secondary to the microbiota-driven inflammatory response and is mediated by tissue-resident immune cells and their products. Combinations of such models have also been used to further our understanding of intestinal pathologies. Thus, *C. rodentium* and *Fusobacterium nucleatum* infection of *Apc* mutant mice enhances the recruitment of tumor-infiltrating myeloid cells, thereby establishing an inflammatory environment that favors tumor progression [177, 178]. Similarly, *Helicobacter hepaticus* amplifies inflammation-driven tumorigenesis in IL10-deficient mice exposed to the mutagen azoxymethane [179]. Conversely, probiotic bactreria and fermented milk create a nonpermissive environment for colitogenic *Enterobacteriaceae* in T-bet/Rag2 mutant mice [180].

These models have highlighted diverse functions of microbial-sensing pattern recognition receptors (PRRs) in epithelial *versus* innate immune cells [181]. Among them, Toll-like (TLR) and NOD-like (NLR) receptors have attracted particular attention because of the association of *NOD2*, *NLRP3/*inflammasome and *TLR4* genotypic profiles with human IBD. The picture that emerges suggests that upon disruption of the epithelial barrier, PRR signaling in enterocytes is required to restore epithelial architecture and to induce the expression of anti-microbial peptides that dampen microbial effects. NF-κB dominates the protective PRR response [182, 183], while defective PRR signaling in enterocytes results in pathogen outgrowth and exaggerated inflammation. TLR4 is also required for *de novo* expansion of an intestinal cell subpopulation, designated as ISC compartment, in response to microbial products [184]. 

By contrast, PRR signaling in innate immune cells mediates pathogenic inflammatory responses. Unresolved inflammation establishes a microenvironment conducive to malignant transformation of intestinal epithelium undergoing cycles of damage and regeneration, eventually leading to tumorigenesis. For example, TLR5 signaling activation promotes *Salmonella* Typhimurium pathogenesis [185]. Also, PRR signaling in myeloid cells leads to NF-κB-dependent production of the pro-inflammatory cytokine IL-6, which promotes the survival and proliferation of premalignant intestinal epithelial cells through STAT3 pathway activation [30]. Similarly, IL-22 produced by intestinal inflammation-induced innate lymphoid cells is necessary for STAT3 activation in ECs and tumor maintenance [31]. Therefore, PRR signals must be finely balanced to maintain intestinal homeostasis: diminished PRR activation may compromise epithelial barrier function whereas excessive PRR signaling may lead to pathogenic inflammatory responses to microbiota and malignant transformation of epithelial cells. 

Notwithstanding the similarity to humans at the anatomical, histological and genomic level, mouse models are impractical for large-scale studies of the intestinal holo’ome during homeostasis, inflammation and cancer. Experimental limitations aside, there are ethical concerns in using large numbers of mice. Accordingly, simpler organisms, such as fruit flies, provide adjunct systems to identify new genes, pathogenesis mechanisms and drug treatments, wherever the tissues involved are molecularly conserved and cellularly analogous [140].

### A reductionist and a systems biology roadmap to the dynamic intestinal holo’ome

One of the major efforts to systematically reveal the role of microbiota in human health and disease is that of the NIH Human Microbiome Project Consortium, the first phase of which (2008-2012) focused on the diversity and composition of human microbiome. Collectively, these studies demonstrated that the taxonomic composition of the microbiome varies significantly between individuals and could not be used to explain the role of microbiota in health and disease [186]. Studies looking specifically at individuals with inflammatory bowel disease (IBD) showed relative changes in microbial composition, but no simple biomarkers or therapeutic targets were identified. Instead, microbiome metabolic pathways and functions appear to be linked to IBD, prompting the Integrative Human Microbiome Project to gather and analyze in its second phase (2013-2016) personalized, longitudinal multi-omic data on the microbiota, the host, host-microbiota interaction and the role of lifestyle in disease [186]. Similarly to IBD, colon cancer is caused by many factors that act in combination rather than independently. Therefore, it will be pivotal to identify the synergistic activities among the host, its microbes and the systemic and intestinal environment that cause disease. While gene allele combinations, such as between oncogenes and tumor suppressors have been shown to synergize during carcinogenesis [24, 187, 188], combinations between intestinal microbes, the blood and intestinal environment as shaped by lifestyle, and host genetic background may provide a more complete synergistic assessment. 

There are various examples of synergisms between the host genetic background and intestinal microbes, nutrients or metabolites using animal models (Table 1). These suggest mechanisms of disease, but unless validated in additional models and in humans they do not provide proof of disease causation. Using different models e.g. different host strains, diets and microbiota is particularly important because quantitative experimental outcome is context-dependent. Similarly, controlling for the genetic polymorphisms, the microbiome and the habits in human individuals necessitates a personalized medicine approach, because the abundance, distribution and virulence of microbes and metabolites, and gene expression in the intestine differs from person to person and may change over time [92, 126]. The lack of sufficient epidemiological data linking specific microbes, gene alleles and diets to intestinal disease may reflect the lack of clinical studies assessing all of these parameters together in each individual (Figure 1). Multi-omic profiling, such as MOPED (http://www.kolkerlab.org/projects/statistics-bioinformatics/moped), that integrates protein and gene expression databases linking them to genes, their pathways and function is an indicative tool towards this direction. Moreover, microbial multi-omics are fairly well established encouraging their adaptation for intestinal microbiota analysis [189]. Nevertheless, host-microbiota-environment interactions are significantly more challenging requiring customized and sophisticated software, integrated data repositories and standardized sampling of blood, colon biopsy and stool. In this respect the Integrative Human Microbiome Project is expected to pave the way. In essence these studies will identify measurable lifestyle and molecular parameters across different platforms calculating their shift between the young (disease free) and the old (disease-prone) state of the same person. Moreover, follow up studies using genetically defined *Drosophila* and mammalian hosts may assess the role of lifestyle and molecular parameters in facilitating intestinal disease, e.g. by assessing the impact of specific microbes or dysbiotic *vs*. symbiotic microbiota, upon different diets in wild type *vs*. genetically predisposed hosts. Lastly, clinical studies can be designed to assess the effectiveness of therapies against dysbiotic combinations of gene alleles, microbes and environmental factors revealed by model organism studies. 

To streamline the assessment of causation in CRC we propose that human intestinal holo’omes be tested at various levels: 1) the host genome (e.g. SNPs), transcriptome and proteome (from colon biopsies), 2) the mucosal microbial composition (from colon biopsies) and fecal microbiota metagenome and metatranscriptome, 3) the blood secretome (cytokines, metabolites) and 4) intestinal metabolome and proteome (from stool samples) [190] . Assuming that even a subclinical (histologically defined) deregulation predisposes for CRC, holo’ome analysis will need to be done in stool, blood and normal appearing colonic mucosa samples taken at two points in time for each human individual: (a) at a disease-free age, years before the onset of disease or long after disease remission, and (b) at a disease-prone age, upon the onset of subclinical disease (Figure 2). Collected data should be analyzed to: (i) pinpoint detrimental molecular synergisms correlating with intestinal disease e.g. being present in an individual in the disease-prone *vs*. the disease-free state; (ii) determine if detrimental molecular synergisms promote intestinal disease in the appropriately adapted model hosts (i.e. in genetically manipulated flies or mice fed or injected with specific microbes and metabolites), and (iii) targeted elimination of these synergisms e.g. elimination of the dysbiotic microbiota and/or normalization of the blood cytokines or intestinal metabolites should decrease the prevalence of disease in human clinical trials. Prebiotic, probiotic, fecal transplantation and bacteriophage therapies are some of the treatment options potentially available in the foreseeable future [191-193] (Figure 1). Importantly, one should discriminate between the different stages of tumor development (i.e. initiation *vs*. progression and benign *vs*. invasive), because the detrimental synergisms may differ accordingly.

While primarily an open-ended search, measuring CRC-related homo’ome parameters is expected to shed light into key aspects of the disease [194], such as:

a) The age-related and metabolic factors driving chronic low-grade intestinal inflammation, regeneration and tumorigenesis.

b) The relative contributions of local regenerative inflammatory signaling *vs.* systemic inflammatory factors in driving CRC during aging.

c) Interventions (dietary, nutrition or physical exercise) that can modulate or eliminate the sources of chronic inflammation.

d) Senescent stem cell, progenitor and enterocyte responses to intestinal damage and stress.

**Table 1:** Examples of detrimental synergisms between the host genetic background and intestinal microbes, nutrients or metabolites.

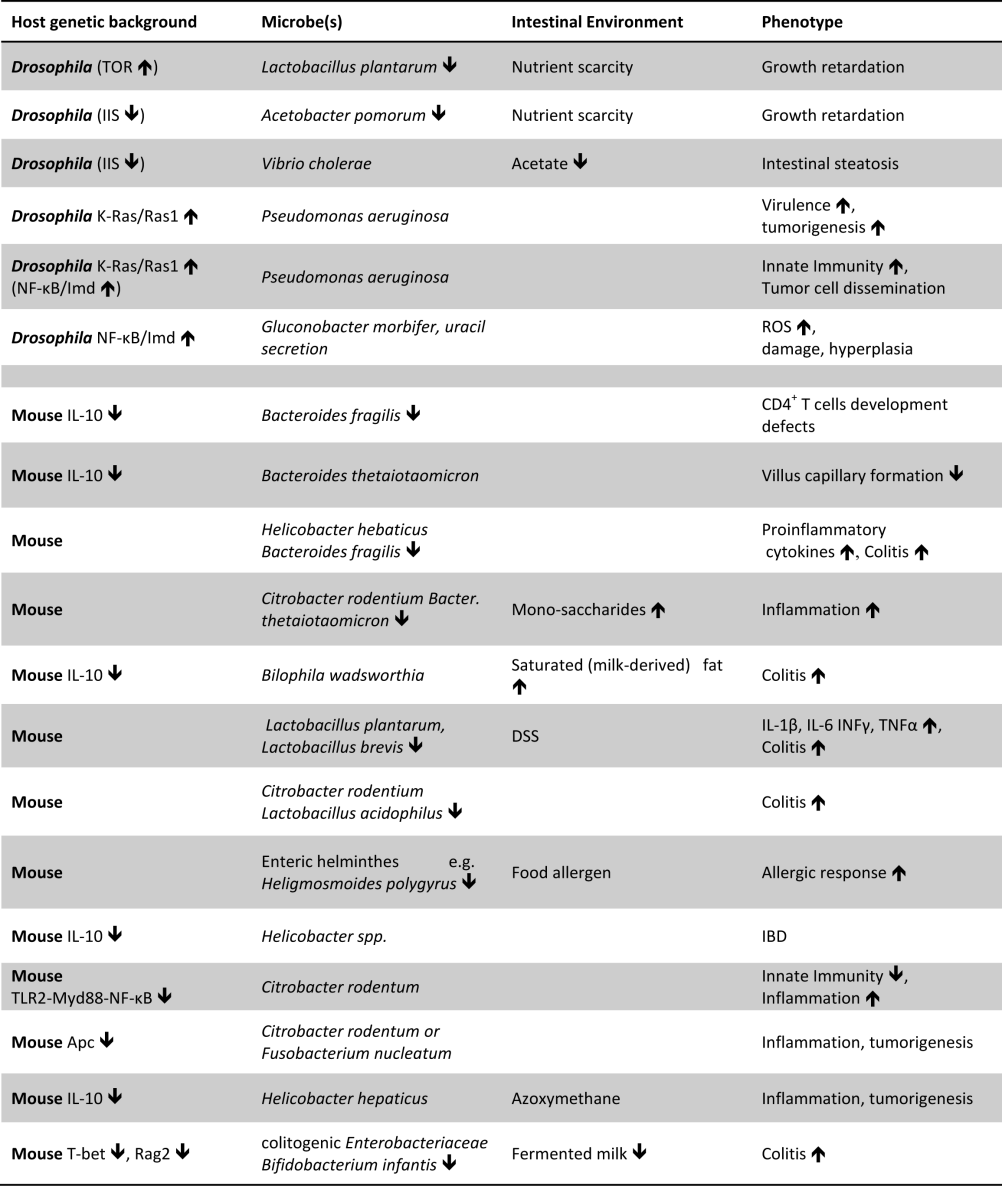

## CONCLUSIONS

*Drosophila* is the simplest model organism sharing substantial human disease gene conservation and intestinal epithelium pathophysiology with humans. Thus, fly in addition to mouse models may guide clinical studies in defining basic parameters of intestinal inflammation and cancer, taking into account the multifaceted and highly complex traits of human intestinal holo’omes. Controlling for the complex genetic, epigenetic, microbiota, lifestyle, gender and age background in future experiments will be critical because in most cases the traits that lead to disease emergence are many, a sum of synergies among gene alleles, microbes and diets that change as we age. Molecular prognosis, diagnosis and treatment options regarding intestinal CRC should be more personalized, and take into consideration synergy within evolving holo’omes of disease-prone *vs.* the disease-free individuals, rather than merely tumor specific genetic/epigenetic markers, as is now customary. At the population level detrimental synergies are likely many and diverse, but our current knowledge is not sufficient to explain the emergence and establishment of CRC. Pinpointing novel factors that drive CRC through longitudinally-changing holo’omes might be laborious and expensive, but necessary to improve CRC prognosis, diagnosis and therapy. 

Such a holo’ome approach might also be applicable to other cancers influenced by our microbiota and lifestyle-related factors according to the “Second-Expert-Report” (World Cancer Research Fund). Unlike cancers that develop at a very young age, such as retinoblastoma and neuroblastoma, which depend heavily on the genetic background of neonates, other malignancies, such as lung, liver and pancreatic cancer, are influenced by our environment, low-grade chronic inflammation and metabolism. Accordingly, intestinal microbiota may affect our inflammatory and metabolic status and may not only impact CRC, but also other cancers developing at an old age. 

## References

[1] Irigaray P, Newby JA, Clapp R, Hardell L, Howard V, Montagnier L, Epstein S, Belpomme D (2007). Lifestyle-related factors and environmental agents causing cancer: an overview. Biomed Pharmacother.

[2] Anand P, Kunnumakkara AB, Sundaram C, Harikumar KB, Tharakan ST, Lai OS, Sung B, Aggarwal BB (2008). Cancer is a preventable disease that requires major lifestyle changes. Pharm Res.

[3] Kuper H, Boffetta P, Adami HO (2002). Tobacco use and cancer causation: association by tumour type. J Intern Med.

[4] Testino G (2011). The burden of cancer attributable to alcohol consumption. Maedica (Buchar).

[5] Parkin DM (2006). The global health burden of infection-associated cancers in the year 2002. Int J Cancer.

[6] Conti CM, Angelucci D, Ferri M, Maccauro G, Caraffa A, Doyle R, Fulcheri M, Cianchetti E (2011). Relationship between cancer and psychology: an updated history. J Biol Regul Homeost Agents.

[7] Calle EE, Rodriguez C, Walker-Thurmond K, Thun MJ (2003). Overweight, obesity, and mortality from cancer in a prospectively studied cohort of U.S. adults. N Engl J Med.

[8] Lee J, Jeon JY, Meyerhardt JA (2015). Diet and lifestyle in survivors of colorectal cancer. Hematol Oncol Clin North Am.

[9] Flint HJ, Scott KP, Louis P, Duncan SH (2012). The role of the gut microbiota in nutrition and health. Nat Rev Gastroenterol Hepatol.

[10] Balkwill F, Mantovani A (2001). Inflammation and cancer: back to Virchow?. Lancet.

[11] Panayidou S, Apidianakis Y (2013). Regenerative inflammation: lessons from Drosophila intestinal epithelium in health and disease. Pathogens.

[12] Ayyaz A, Li H, Jasper H (2015). Haemocytes control stem cell activity in the Drosophila intestine. Nat Cell Biol.

[13] Taniguchi K, Wu LW, Grivennikov SI, de Jong PR, Lian I, Yu FX, Wang K, Ho SB, Boland BS, Chang JT, Sandborn WJ, Hardiman G, Raz E (2015). A gp130-Src-YAP module links inflammation to epithelial regeneration. Nature.

[14] Apidianakis Y, Rahme LG (2011). Drosophila melanogaster as a model for human intestinal infection and pathology. Dis Model Mech.

[15] Kinzler KW, Vogelstein B (1997). Cancer-susceptibility genes. Gatekeepers and caretakers. Nature.

[16] Hanahan D, Weinberg RA (2011). Hallmarks of cancer: the next generation. Cell.

[17] Arcila M, Lau C, Nafa K, Ladanyi M (2011). Detection of KRAS and BRAF mutations in colorectal carcinoma roles for high-sensitivity locked nucleic acid-PCR sequencing and broad-spectrum mass spectrometry genotyping. J Mol Diagn.

[18] de Caestecker MP, Piek E, Roberts AB (2000). Role of transforming growth factor-beta signaling in cancer. J Natl Cancer Inst.

[19] Losi L, Bouzourene H, Benhattar J (2007). Loss of Smad4 expression predicts liver metastasis in human colorectal cancer. Oncol Rep.

[20] Schwitalla S, Ziegler PK, Horst D, Becker V, Kerle I, Begus-Nahrmann Y, Lechel A, Rudolph KL, Langer R, Slotta-Huspenina J, Bader FG, Prazeres da Costa O, Neurath MF (2013). Loss of p53 in enterocytes generates an inflammatory microenvironment enabling invasion and lymph node metastasis of carcinogen-induced colorectal tumors. Cancer Cell.

[21] Song MS, Carracedo A, Salmena L, Song SJ, Egia A, Malumbres M, Pandolfi PP (2011). Nuclear PTEN regulates the APC-CDH1 tumor-suppressive complex in a phosphatase-independent manner. Cell.

[22] Kato H, Semba S, Miskad UA, Seo Y, Kasuga M, Yokozaki H (2004). High expression of PRL-3 promotes cancer cell motility and liver metastasis in human colorectal cancer: a predictive molecular marker of metachronous liver and lung metastases. Clin Cancer Res.

[23] Martorell Ò, Merlos-Suárez A, Campbell K, Barriga FM, Christov CP, Miguel-Aliaga I, Batlle E, Casanova J, Casali A (2014). Conserved mechanisms of tumorigenesis in the Drosophila adult midgut. PLoS One.

[24] Wu M, Pastor-Pareja JC, Xu T (2010). Interaction between Ras(V12) and scribbled clones induces tumour growth and invasion. Nature.

[25] Doggett K, Grusche FA, Richardson HE, Brumby AM (2011). Loss of the Drosophila cell polarity regulator Scribbled promotes epithelial tissue overgrowth and cooperation with oncogenic Ras-Raf through impaired Hippo pathway signaling. BMC Dev Biol.

[26] Ogura Y, Bonen DK, Inohara N, Nicolae DL, Chen FF, Ramos R, Britton H, Moran T, Karaliuskas R, Duerr RH, Achkar JP, Brant SR, Bayless TM (2001). A frameshift mutation in NOD2 associated with susceptibility to Crohn’s disease. Nature.

[27] Franchimont D, Vermeire S, El Housni H, Pierik M, Van Steen K, Gustot T, Quertinmont E, Abramowicz M, Van Gossum A, Devière J, Rutgeerts P (2004). Deficient host-bacteria interactions in inflammatory bowel disease? The toll-like receptor (TLR)-4 Asp299gly polymorphism is associated with Crohn’s disease and ulcerative colitis. Gut.

[28] Cadwell K, Patel KK, Maloney NS, Liu TC, Ng AC, Storer CE, Head RD, Xavier R, Stappenbeck TS, Virgin HW (2010). Virus-plus-susceptibility gene interaction determines Crohn’s disease gene Atg16L1 phenotypes in intestine. Cell.

[29] Rad R, Dossumbekova A, Neu B, Lang R, Bauer S, Saur D, Gerhard M, Prinz C (2004). Cytokine gene polymorphisms influence mucosal cytokine expression, gastric inflammation, and host specific colonisation during Helicobacter pylori infection. Gut.

[30] Grivennikov S, Karin E, Terzic J, Mucida D, Yu GY, Vallabhapurapu S, Scheller J, Rose-John S, Cheroutre H, Eckmann L, Karin M (2009). IL-6 and Stat3 are required for survival of intestinal epithelial cells and development of colitis-associated cancer. Cancer Cell.

[31] Kirchberger S, Royston DJ, Boulard O, Thornton E, Franchini F, Szabady RL, Harrison O, Powrie F (2013). Innate lymphoid cells sustain colon cancer through production of interleukin-22 in a mouse model. J Exp Med.

[32] Levine B, Mizushima N, Virgin HW (2011). Autophagy in immunity and inflammation. Nature.

[33] Jones RM, Luo L, Ardita CS, Richardson AN, Kwon YM, Mercante JW, Alam A, Gates CL, Wu H, Swanson PA, Lambeth JD, Denning PW, Neish AS (2013). Symbiotic lactobacilli stimulate gut epithelial proliferation via Nox-mediated generation of reactive oxygen species. EMBO J.

[34] Ayyaz A, Jasper H (2013). Intestinal inflammation and stem cell homeostasis in aging Drosophila melanogaster. Front Cell Infect Microbiol.

[35] Kim SH, Lee WJ (2014). Role of DUOX in gut inflammation: lessons from Drosophila model of gut-microbiota interactions. Front Cell Infect Microbiol.

[36] Ferlitsch M, Reinhart K, Pramhas S, Wiener C, Gal O, Bannert C, Hassler M, Kozbial K, Dunkler D, Trauner M, Weiss W (2011). Sex-specific prevalence of adenomas, advanced adenomas, and colorectal cancer in individuals undergoing screening colonoscopy. JAMA.

[37] Amos-Landgraf JM, Heijmans J, Wielenga MC, Dunkin E, Krentz KJ, Clipson L, Ederveen AG, Groothuis PG, Mosselman S, Muncan V, Hommes DW, Shedlovsky A, Dove WF, van den Brink GR (2014). Sex disparity in colonic adenomagenesis involves promotion by male hormones, not protection by female hormones. Proc Natl Acad Sci U S A.

[38] Saleiro D, Murillo G, Benya RV, Bissonnette M, Hart J, Mehta RG (2012). Estrogen receptor-β protects against colitis-associated neoplasia in mice. Int J Cancer.

[39] Cook LC, Hillhouse AE, Myles MH, Lubahn DB, Bryda EC, Davis JW, Franklin CL (2014). The role of estrogen signaling in a mouse model of inflammatory bowel disease: a Helicobacter hepaticus model. PLoS One.

[40] Kane SV, Reddy D (2008). Hormonal replacement therapy after menopause is protective of disease activity in women with inflammatory bowel disease. Am J Gastroenterol.

[41] Coppedè F (2014). The role of epigenetics in colorectal cancer. Expert Rev Gastroenterol Hepatol.

[42] Akhtar-Zaidi B, Cowper-Sal-lari R, Corradin O, Saiakhova A, Bartels CF, Balasubramanian D, Myeroff L, Lutterbaugh J, Jarrar A, Kalady MF, Willis J, Moore JH, Tesar PJ (2012). Epigenomic enhancer profiling defines a signature of colon cancer. Science.

[43] Foran E, Garrity-Park MM, Mureau C, Newell J, Smyrk TC, Limburg PJ, Egan LJ (2010). Upregulation of DNA methyltransferase-mediated gene silencing, anchorage-independent growth, and migration of colon cancer cells by interleukin-6. Mol Cancer Res.

[44] Lee H, Zhang P, Herrmann A, Yang C, Xin H, Wang Z, Hoon DS, Forman SJ, Jove R, Riggs AD, Yu H (2012). Acetylated STAT3 is crucial for methylation of tumor-suppressor gene promoters and inhibition by resveratrol results in demethylation. Proc Natl Acad Sci U S A.

[45] Daniluk J, Liu Y, Deng D, Chu J, Huang H, Gaiser S, Cruz-Monserrate Z, Wang H, Ji B, Logsdon CD (2012). An NF-κB pathway-mediated positive feedback loop amplifies Ras activity to pathological levels in mice. J Clin Invest.

[46] Zwiers A, Kraal L, van de Pouw Kraan TC, Wurdinger T, Bouma G, Kraal G (2012). Cutting edge: a variant of the IL-23R gene associated with inflammatory bowel disease induces loss of microRNA regulation and enhanced protein production. J Immunol.

[47] Eckburg PB, Bik EM, Bernstein CN, Purdom E, Dethlefsen L, Sargent M, Gill SR, Nelson KE, Relman DA (2005). Diversity of the human intestinal microbial flora. Science.

[48] Gu S, Chen D, Zhang JN, Lv X, Wang K, Duan LP, Nie Y, Wu XL (2013). Bacterial community mapping of the mouse gastrointestinal tract. PLoS One.

[49] Mackie RI, Sghir A, Gaskins HR (1999). Developmental microbial ecology of the neonatal gastrointestinal tract. Am J Clin Nutr.

[50] Dethlefsen L, McFall-Ngai M, Relman DA (2007). An ecological and evolutionary perspective on human-microbe mutualism and disease. Nature.

[51] Ding T, Schloss PD (2014). Dynamics and associations of microbial community types across the human body. Nature.

[52] Lombardi P, Goldin B, Boutin E, Gorbach SL (1978). Metabolism of androgens and estrogens by human fecal microorganisms. J Steroid Biochem.

[53] Ridlon JM, Ikegawa S, Alves JM, Zhou B, Kobayashi A, Iida T, Mitamura K, Tanabe G, Serrano M, De Guzman A, Cooper P, Buck GA, Hylemon PB (2013). Clostridium scindens: a human gut microbe with a high potential to convert glucocorticoids into androgens. J Lipid Res.

[54] Markle JG, Frank DN, Mortin-Toth S, Robertson CE, Feazel LM, Rolle-Kampczyk U, von Bergen M, McCoy KD, Macpherson AJ, Danska JS (2013). Sex differences in the gut microbiome drive hormone-dependent regulation of autoimmunity. Science.

[55] Chandler JA, Lang JM, Bhatnagar S, Eisen JA, Kopp A (2011). Bacterial communities of diverse Drosophila species: ecological context of a host-microbe model system. PLoS Genet.

[56] Broderick NA, Buchon N, Lemaitre B (2014). Microbiota-induced changes in drosophila melanogaster host gene expression and gut morphology. MBio.

[57] Stein RR, Bucci V, Toussaint NC, Buffie CG, Rätsch G, Pamer EG, Sander C, Xavier JB (2013). Ecological modeling from time-series inference: insight into dynamics and stability of intestinal microbiota. PLOS Comput Biol.

[58] Lee KA, Lee WJ (2014). Drosophila as a model for intestinal dysbiosis and chronic inflammatory diseases. Dev Comp Immunol.

[59] Buffie CG, Pamer EG (2013). Microbiota-mediated colonization resistance against intestinal pathogens. Nat Rev Immunol.

[60] Ryu JH, Kim SH, Lee HY, Bai JY, Nam YD, Bae JW, Lee DG, Shin SC, Ha EM, Lee WJ (2008). Innate immune homeostasis by the homeobox gene caudal and commensal-gut mutualism in Drosophila. Science.

[61] Flint HJ, Bayer EA, Rincon MT, Lamed R, White BA (2008). Polysaccharide utilization by gut bacteria: potential for new insights from genomic analysis. Nat Rev Microbiol.

[62] Maslowski KM, Vieira AT, Ng A, Kranich J, Sierro F, Yu D, Schilter HC, Rolph MS, Mackay F, Artis D, Xavier RJ, Teixeira MM, Mackay CR (2009). Regulation of inflammatory responses by gut microbiota and chemoattractant receptor GPR43. Nature.

[63] Storelli G, Defaye A, Erkosar B, Hols P, Royet J, Leulier F (2011). Lactobacillus plantarum promotes Drosophila systemic growth by modulating hormonal signals through TOR-dependent nutrient sensing. Cell Metab.

[64] Shin SC, Kim SH, You H, Kim B, Kim AC, Lee KA, Yoon JH, Ryu JH, Lee WJ (2011). Drosophila microbiome modulates host developmental and metabolic homeostasis via insulin signaling. Science.

[65] Hang S, Purdy AE, Robins WP, Wang Z, Mandal M, Chang S, Mekalanos JJ, Watnick PI (2014). The acetate switch of an intestinal pathogen disrupts host insulin signaling and lipid metabolism. Cell Host Microbe.

[66] Kelly D, Campbell JI, King TP, Grant G, Jansson EA, Coutts AG, Pettersson S, Conway S (2004). Commensal anaerobic gut bacteria attenuate inflammation by regulating nuclear-cytoplasmic shuttling of PPAR-γ and RelA. Nat Immunol.

[67] Mazmanian SK, Liu CH, Tzianabos AO, Kasper DL (2005). An immunomodulatory molecule of symbiotic bacteria directs maturation of the host immune system. Cell.

[68] Christensen HR, Frøkiaer H, Pestka JJ (2002). Lactobacilli differentially modulate expression of cytokines and maturation surface markers in murine dendritic cells. J Immunol.

[69] Fink LN, Zeuthen LH, Christensen HR, Morandi B, Frøkiaer H, Ferlazzo G (2007). Distinct gut-derived lactic acid bacteria elicit divergent dendritic cell-mediated NK cell responses. Int Immunol.

[70] Panayidou S, Ioannidou E, Apidianakis Y (2014). Human pathogenic bacteria, fungi, and viruses in Drosophila: disease modeling, lessons, and shortcomings. Virulence.

[71] Hooper LV (2004). Bacterial contributions to mammalian gut development. Trends Microbiol.

[72] Chung H, Kasper DL (2010). Microbiota-stimulated immune mechanisms to maintain gut homeostasis. Curr Opin Immunol.

[73] Hooper LV, Stappenbeck TS, Hong CV, Gordon JI (2003). Angiogenins: a new class of microbicidal proteins involved in innate immunity. Nat Immunol.

[74] Chung H, Pamp SJ, Hill JA, Surana NK, Edelman SM, Troy EB, Reading NC, Villablanca EJ, Wang S, Mora JR, Umesaki Y, Mathis D, Benoist C (2012). Gut immune maturation depends on colonization with a host-specific microbiota. Cell.

[75] Suzuki K, Meek B, Doi Y, Muramatsu M, Chiba T, Honjo T, Fagarasan S (2004). Aberrant expansion of segmented filamentous bacteria in IgA-deficient gut. Proc Natl Acad Sci U S A.

[76] Round JL, Mazmanian SK (2009). The gut microbiota shapes intestinal immune responses during health and disease. Nat Rev Immunol.

[77] Bäckhed F, Ding H, Wang T, Hooper LV, Koh GY, Nagy A, Semenkovich CF, Gordon JI (2004). The gut microbiota as an environmental factor that regulates fat storage. Proc Natl Acad Sci U S A.

[78] Xu J, Gordon JI (2003). Honor thy symbionts. Proc Natl Acad Sci U S A.

[79] Mazmanian SK, Round JL, Kasper DL (2008). A microbial symbiosis factor prevents intestinal inflammatory disease. Nature.

[80] Clarridge JE 3rd (2004). Impact of 16S rRNA gene sequence analysis for identification of bacteria on clinical microbiology and infectious diseases. Clin Microbiol Rev.

[81] Manichanh C, Rigottier-Gois L, Bonnaud E, Gloux K, Pelletier E, Frangeul L, Nalin R, Jarrin C, Chardon P, Marteau P, Roca J, Dore J (2006). Reduced diversity of faecal microbiota in Crohn’s disease revealed by a metagenomic approach. Gut.

[82] Boleij A, Tjalsma H (2013). The itinerary of Streptococcus gallolyticus infection in patients with colonic malignant disease. Lancet Infect Dis.

[83] Toprak NU, Yagci A, Gulluoglu BM, Akin ML, Demirkalem P, Celenk T, Soyletir G (2006). A possible role of Bacteroides fragilis enterotoxin in the aetiology of colorectal cancer. Clin Microbiol Infect.

[84] Swidsinski A, Khilkin M, Kerjaschki D, Schreiber S, Ortner M, Weber J, Lochs H (1998). Association between intraepithelial Escherichia coli and colorectal cancer. Gastroenterology.

[85] Castellarin M, Warren RL, Freeman JD, Dreolini L, Krzywinski M, Strauss J, Barnes R, Watson P, Allen-Vercoe E, Moore RA, Holt RA (2012). Fusobacterium nucleatum infection is prevalent in human colorectal carcinoma. Genome Res.

[86] Sartor RB (2008). Microbial influences in inflammatory bowel diseases. Gastroenterology.

[87] Lysenko ES, Ratner AJ, Nelson AL, Weiser JN (2005). The role of innate immune responses in the outcome of interspecies competition for colonization of mucosal surfaces. PLoS Pathog.

[88] Garrett WS, Lord GM, Punit S, Lugo-Villarino G, Mazmanian SK, Ito S, Glickman JN, Glimcher LH (2007). Communicable ulcerative colitis induced by T-bet deficiency in the innate immune system. Cell.

[89] Guo L, Karpac J, Tran SL, Jasper H (2014). PGRP-SC2 promotes gut immune homeostasis to limit commensal dysbiosis and extend lifespan. Cell.

[90] Zackular JP, Baxter NT, Iverson KD, Sadler WD, Petrosino JF, Chen GY, Schloss PD (2013). The gut microbiome modulates colon tumorigenesis. MBio.

[91] Arthur JC, Perez-Chanona E, Mühlbauer M, Tomkovich S, Uronis JM, Fan TJ, Campbell BJ, Abujamel T, Dogan B, Rogers AB, Rhodes JM, Stintzi A, Simpson KW (2012). Intestinal inflammation targets cancer-inducing activity of the microbiota. Science.

[92] Tjalsma H, Boleij A, Marchesi JR, Dutilh BE (2012). A bacterial driver-passenger model for colorectal cancer: beyond the usual suspects. Nat Rev Microbiol.

[93] Lahti L, Salojärvi J, Salonen A, Scheffer M, de Vos WM (2014). Tipping elements in the human intestinal ecosystem. Nat Commun.

[94] Howlader N, Noone A, Krapcho M, Garshell J, Miller D, Altekruse S, Kosary C, Yu M, Ruhl J, Tatalovich Z, Mariotto A, Lewis D, Chen H (2012). SEER Cancer Statistics Review, 1975-2009 (Vintage 2009 Populations).

[95] David LA, Maurice CF, Carmody RN, Gootenberg DB, Button JE, Wolfe BE, Ling AV, Devlin AS, Varma Y, Fischbach MA, Biddinger SB, Dutton RJ, Turnbaugh PJ (2014). Diet rapidly and reproducibly alters the human gut microbiome. Nature.

[96] Turnbaugh PJ, Ridaura VK, Faith JJ, Rey FE, Knight R, Gordon JI (2009). The effect of diet on the human gut microbiome: a metagenomic analysis in humanized gnotobiotic mice. Sci Transl Med.

[97] Kamada N, Kim YG, Sham HP, Vallance BA, Puente JL, Martens EC, Núñez G (2012). Regulated virulence controls the ability of a pathogen to compete with the gut microbiota. Science.

[98] Wong AC, Dobson AJ, Douglas AE (2014). Gut microbiota dictates the metabolic response of Drosophila to diet. J Exp Biol.

[99] Kapsetaki SE, Tzelepis I, Avgousti K, Livadaras I, Garantonakis N, Varikou K, Apidianakis Y (2014). The bacterial metabolite 2-aminoacetophenone promotes association of pathogenic bacteria with flies. Nat Commun.

[100] Bolnick DI, Snowberg LK, Hirsch PE, Lauber CL, Org E, Parks B, Lusis AJ, Knight R, Caporaso JG, Svanbäck R (2014). Individual diet has sex-dependent effects on vertebrate gut microbiota. Nat Commun.

[101] Devkota S, Wang Y, Musch MW, Leone V, Fehlner-Peach H, Nadimpalli A, Antonopoulos DA, Jabri B, Chang EB (2012). Dietary-fat-induced taurocholic acid promotes pathobiont expansion and colitis in Il10-/- mice. Nature.

[102] Maslowski KM, Mackay CR (2011). Diet, gut microbiota and immune responses. Nat Immunol.

[103] Smith PM, Howitt MR, Panikov N, Michaud M, Gallini CA, Bohlooly-Y M, Glickman JN, Garrett WS (2013). The microbial metabolites, short-chain fatty acids, regulate colonic Treg cell homeostasis. Science.

[104] Furusawa Y, Obata Y, Fukuda S, Endo TA, Nakato G, Takahashi D, Nakanishi Y, Uetake C, Kato K, Kato T, Takahashi M, Fukuda NN, Murakami S (2013). Commensal microbe-derived butyrate induces the differentiation of colonic regulatory T cells. Nature.

[105] Arpaia N, Campbell C, Fan X, Dikiy S, van der Veeken J, deRoos P, Liu H, Cross JR, Pfeffer K, Coffer PJ, Rudensky AY (2013). Metabolites produced by commensal bacteria promote peripheral regulatory T-cell generation. Nature.

[106] Issa JP, Ahuja N, Toyota M, Bronner MP, Brentnall TA (2001). Accelerated age-related CpG island methylation in ulcerative colitis. Cancer Res.

[107] Tahara T, Yamamoto E, Suzuki H, Maruyama R, Chung W, Garriga J, Jelinek J, Yamano HO, Sugai T, An B, Shureiqi I, Toyota M, Kondo Y (2014). Fusobacterium in colonic flora and molecular features of colorectal carcinoma. Cancer Res.

[108] Williams CD, Satia JA, Adair LS, Stevens J, Galanko J, Keku TO, Sandler RS (2010). Associations of red meat, fat, and protein intake with distal colorectal cancer risk. Nutr Cancer.

[109] Sears CL, Garrett WS (2014). Microbes, microbiota, and colon cancer. Cell Host Microbe.

[110] Gill CI, Rowland IR (2002). Diet and cancer: assessing the risk. Br J Nutr.

[111] Mühlbauer M, Allard B, Bosserhoff AK, Kiessling S, Herfarth H, Rogler G, Schölmerich J, Jobin C, Hellerbrand C (2004). Differential effects of deoxycholic acid and taurodeoxycholic acid on NF-kappa B signal transduction and IL-8 gene expression in colonic epithelial cells. Am J Physiol Gastrointest Liver Physiol.

[112] Da Silva M, Jaggers GK, Verstraeten SV, Erlejman AG, Fraga CG, Oteiza PI (2012). Large procyanidins prevent bile-acid-induced oxidant production and membrane-initiated ERK1/2, p38, and Akt activation in Caco-2 cells. Free Radic Biol Med.

[113] Park J, Floch MH (2007). Prebiotics, probiotics, and dietary fiber in gastrointestinal disease. Gastroenterol Clin North Am.

[114] Nell S, Suerbaum S, Josenhans C (2010). The impact of the microbiota on the pathogenesis of IBD: lessons from mouse infection models. Nat Rev Microbiol.

[115] Lee HS, Han SY, Bae EA, Huh CS, Ahn YT, Lee JH, Kim DH (2008). Lactic acid bacteria inhibit proinflammatory cytokine expression and bacterial glycosaminoglycan degradation activity in dextran sulfate sodium-induced colitic mice. Int Immunopharmacol.

[116] Chen CC, Louie S, Shi HN, Walker WA (2005). Preinoculation with the probiotic Lactobacillus acidophilus early in life effectively inhibits murine Citrobacter rodentium colitis. Pediatr Res.

[117] Apidianakis Y, Pitsouli C, Perrimon N, Rahme L (2009). Synergy between bacterial infection and genetic predisposition in intestinal dysplasia. Proc Natl Acad Sci U S A.

[118] Buchon N, Broderick NA, Chakrabarti S, Lemaitre B (2009). Invasive and indigenous microbiota impact intestinal stem cell activity through multiple pathways in Drosophila. Genes Dev.

[119] Jiang H, Patel PH, Kohlmaier A, Grenley MO, McEwen DG, Edgar BA (2009). Cytokine/Jak/Stat signaling mediates regeneration and homeostasis in the Drosophila midgut. Cell.

[120] Zhu Y, Michelle Luo T, Jobin C, Young HA (2011). Gut microbiota and probiotics in colon tumorigenesis. Cancer Lett.

[121] Zitvogel L, Galluzzi L, Viaud S, Vétizou M, Daillère R, Merad M, Kroemer G (2015). Cancer and the gut microbiota: an unexpected link. Sci Transl Med.

[122] De Filippo C, Cavalieri D, Di Paola M, Ramazzotti M, Poullet JB, Massart S, Collini S, Pieraccini G, Lionetti P (2010). Impact of diet in shaping gut microbiota revealed by a comparative study in children from Europe and rural Africa. Proc Natl Acad Sci U S A.

[123] Looft T, Allen HK (2012). Collateral effects of antibiotics on mammalian gut microbiomes. Gut Microbes.

[124] Wohlgemuth S, Haller D, Blaut M, Loh G (2009). Reduced microbial diversity and high numbers of one single Escherichia coli strain in the intestine of colitic mice. Environ Microbiol.

[125] Flanagan JL, Brodie EL, Weng L, Lynch SV, Garcia O, Brown R, Hugenholtz P, DeSantis TZ, Andersen GL, Wiener-Kronish JP, Bristow J (2007). Loss of bacterial diversity during antibiotic treatment of intubated patients colonized with Pseudomonas aeruginosa. J Clin Microbiol.

[126] Markou P, Apidianakis Y (2014). Pathogenesis of intestinal Pseudomonas aeruginosa infection in patients with cancer. Front Cell Infect Microbiol.

[127] Papoff P, Ceccarelli G, d’Ettorre G, Cerasaro C, Caresta E, Midulla F, Moretti C (2012). Gut microbial translocation in critically ill children and effects of supplementation with pre- and pro biotics. Int J Microbiol.

[128] Löfmark S, Jernberg C, Jansson JK, Edlund C (2006). Clindamycin-induced enrichment and long-term persistence of resistant Bacteroides spp. and resistance genes. J Antimicrob Chemother.

[129] Sjölund M, Tano E, Blaser MJ, Andersson DI, Engstrand L (2005). Persistence of resistant Staphylococcus epidermidis after single course of clarithromycin. Emerg Infect Dis.

[130] Blum JE, Fischer CN, Miles J, Handelsman J (2013). Frequent replenishment sustains the beneficial microbiome of Drosophila melanogaster. MBio.

[131] Strachan DP (1989). Hay fever, hygiene, and household size. BMJ.

[132] Gent AE, Hellier MD, Grace RH, Swarbrick ET, Coggon D (1994). Inflammatory bowel disease and domestic hygiene in infancy. Lancet.

[133] Wold AE (1998). The hygiene hypothesis revised: is the rising frequency of allergy due to changes in the intestinal flora?. Allergy.

[134] Bach JF (2002). The effect of infections on susceptibility to autoimmune and allergic diseases. N Engl J Med.

[135] Macpherson AJ, Harris NL (2004). Interactions between commensal intestinal bacteria and the immune system. Nat Rev Immunol.

[136] East R (2013). Microbiome: soil science comes to life. Nature.

[137] Bashir ME, Andersen P, Fuss IJ, Shi HN, Nagler-Anderson C (2002). An enteric helminth infection protects against an allergic response to dietary antigen. J Immunol.

[138] Longo VD, Finch CE (2003). Evolutionary medicine: from dwarf model systems to healthy centenarians?. Science.

[139] Kostic AD, Howitt MR, Garrett WS (2013). Exploring host-microbiota interactions in animal models and humans. Genes Dev.

[140] Tzelepis I, Kapsetaki SE, Panayidou S, Apidianakis Y (2013). Drosophila melanogaster: a first step and a stepping-stone to anti-infectives. Curr Opin Pharmacol.

[141] Skaer H, Bate M, Arias A (1993). The Alimentary Canal. The development of Drosophila melanogaster.

[142] Micchelli CA, Perrimon N (2006). Evidence that stem cells reside in the adult Drosophila midgut epithelium. Nature.

[143] Ohlstein B, Spradling A (2006). The adult Drosophila posterior midgut is maintained by pluripotent stem cells. Nature.

[144] Li L, Clevers H (2010). Coexistence of quiescent and active adult stem cells in mammals. Science.

[145] Buchon N, Osman D, David FP, Fang HY, Boquete JP, Deplancke B, Lemaitre B (2013). Morphological and molecular characterization of adult midgut compartmentalization in Drosophila. Cell Rep.

[146] Marianes A, Spradling AC (2013). Physiological and stem cell compartmentalization within the Drosophila midgut. Elife.

[147] Fox DT, Spradling AC (2009). The Drosophila hindgut lacks constitutively active adult stem cells but proliferates in response to tissue damage. Cell Stem Cell.

[148] Charroux B, Royet J (2012). Gut-microbiota interactions in non-mammals: what can we learn from Drosophila?. Semin Immunol.

[149] Cordero JB, Stefanatos RK, Scopelliti A, Vidal M, Sansom OJ (2012). Inducible progenitor-derived Wingless regulates adult midgut regeneration in Drosophila. EMBO J.

[150] de Navascués J, Perdigoto CN, Bian Y, Schneider MH, Bardin AJ, Martínez-Arias A, Simons BD (2012). Drosophila midgut homeostasis involves neutral competition between symmetrically dividing intestinal stem cells. EMBO J.

[151] Kux K, Pitsouli C (2014). Tissue communication in regenerative inflammatory signaling: lessons from the fly gut. Front Cell Infect Microbiol.

[152] Christofi T, Apidianakis Y (2013). Drosophila and the hallmarks of cancer. Adv Biochem Eng Biotechnol.

[153] Gonzalez C (2013). Drosophila melanogaster: a model and a tool to investigate malignancy and identify new therapeutics. Nat Rev Cancer.

[154] Reiter LT, Potocki L, Chien S, Gribskov M, Bier E (2001). A systematic analysis of human disease-associated gene sequences in Drosophila melanogaster. Genome Res.

[155] Ellisen LW, Bird J, West DC, Soreng AL, Reynolds TC, Smith SD, Sklar J (1991). TAN-1, the human homolog of the Drosophila notch gene, is broken by chromosomal translocations in T lymphoblastic neoplasms. Cell.

[156] Harvey K, Tapon N (2007). The Salvador-Warts-Hippo pathway - an emerging tumour-suppressor network. Nat Rev Cancer.

[157] Bina S, Zeidler M, Stephanou Anastasis (2009). JAK/STAT pathway signalling in Drosophila melanogaster. JAK-STAT Pathway in Disease.

[158] Zeng X, Singh SR, Hou D, Hou SX (2010). Tumor suppressors Sav/Scrib and oncogene Ras regulate stem-cell transformation in adult Drosophila malpighian tubules. J Cell Physiol.

[159] Ohsawa S, Sato Y, Enomoto M, Nakamura M, Betsumiya A, Igaki T (2012). Mitochondrial defect drives non-autonomous tumour progression through Hippo signalling in Drosophila. Nature.

[160] Lin G, Xu N, Xi R (2008). Paracrine Wingless signalling controls self-renewal of Drosophila intestinal stem cells. Nature.

[161] Biteau B, Hochmuth CE, Jasper H (2008). JNK activity in somatic stem cells causes loss of tissue homeostasis in the aging Drosophila gut. Cell Stem Cell.

[162] Choi NH, Kim JG, Yang DJ, Kim YS, Yoo MA (2008). Age-related changes in Drosophila midgut are associated with PVF2, a PDGF/VEGF-like growth factor. Aging Cell.

[163] Cronin SJ, Nehme NT, Limmer S, Liegeois S, Pospisilik JA, Schramek D, Leibbrandt A, Simoes RM, Gruber S, Puc U, Ebersberger I, Zoranovic T, Neely GG (2009). Genome-wide RNAi screen identifies genes involved in intestinal pathogenic bacterial infection. Science.

[164] Buchon N, Broderick NA, Poidevin M, Pradervand S, Lemaitre B (2009). Drosophila intestinal response to bacterial infection: activation of host defense and stem cell proliferation. Cell Host Microbe.

[165] Amcheslavsky A, Jiang J, Ip YT (2009). Tissue damage-induced intestinal stem cell division in Drosophila. Cell Stem Cell.

[166] Ren F, Wang B, Yue T, Yun EY, Ip YT, Jiang J (2010). Hippo signaling regulates Drosophila intestine stem cell proliferation through multiple pathways. Proc Natl Acad Sci U S A.

[167] Salomon RN, Jackson FR (2008). Tumors of testis and midgut in aging flies. Fly (Austin).

[168] Korgaonkar A, Trivedi U, Rumbaugh KP, Whiteley M (2013). Community surveillance enhances Pseudomonas aeruginosa virulence during polymicrobial infection. Proc Natl Acad Sci U S A.

[169] Bangi E, Pitsouli C, Rahme LG, Cagan R, Apidianakis Y (2012). Immune response to bacteria induces dissemination of Ras-activated Drosophila hindgut cells. EMBO Rep.

[170] Christofi T, Apidianakis Y (2013). Ras-oncogenic Drosophila hindgut but not midgut cells use an inflammation-like program to disseminate to distant sites. Gut Microbes.

[171] Wang L, Kounatidis I, Ligoxygakis P (2014). Drosophila as a model to study the role of blood cells in inflammation, innate immunity and cancer. Front Cell Infect Microbiol.

[172] Pastor-Pareja JC, Wu M, Xu T (2008). An innate immune response of blood cells to tumors and tissue damage in Drosophila. Dis Model Mech.

[173] Cordero JB, Macagno JP, Stefanatos RK, Strathdee KE, Cagan RL, Vidal M (2010). Oncogenic Ras diverts a host TNF tumor suppressor activity into tumor promoter. Dev Cell.

[174] Waterston RH, Lindblad-Toh K, Birney E, Rogers J, Abril JF, Agarwal P, Agarwala R, Ainscough R, Alexandersson M, An P, Antonarakis SE, Attwood J, Baertsch R, and Mouse Genome Sequencing Consortium (2002). Initial sequencing and comparative analysis of the mouse genome. Nature.

[175] Peters LL, Robledo RF, Bult CJ, Churchill GA, Paigen BJ, Svenson KL (2007). The mouse as a model for human biology: a resource guide for complex trait analysis. Nat Rev Genet.

[176] Gkouskou KK, Deligianni C, Tsatsanis C, Eliopoulos AG (2014). The gut microbiota in mouse models of inflammatory bowel disease. Front Cell Infect Microbiol.

[177] Newman JV, Kosaka T, Sheppard BJ, Fox JG, Schauer DB (2001). Bacterial infection promotes colon tumorigenesis in Apc(Min/+) mice. J Infect Dis.

[178] Kostic AD, Chun E, Robertson L, Glickman JN, Gallini CA, Michaud M, Clancy TE, Chung DC, Lochhead P, Hold GL, El-Omar EM, Brenner D, Fuchs CS (2013). Fusobacterium nucleatum potentiates intestinal tumorigenesis and modulates the tumor-immune microenvironment. Cell Host Microbe.

[179] Nagamine CM, Rogers AB, Fox JG, Schauer DB (2008). Helicobacter hepaticus promotes azoxymethane-initiated colon tumorigenesis in BALB/c-IL10-deficient mice. Int J Cancer.

[180] Veiga P, Gallini CA, Beal C, Michaud M, Delaney ML, DuBois A, Khlebnikov A, van Hylckama Vlieg JE, Punit S, Glickman JN, Onderdonk A, Glimcher LH, Garrett WS (2010). Bifidobacterium animalis subsp. lactis fermented milk product reduces inflammation by altering a niche for colitogenic microbes. Proc Natl Acad Sci U S A.

[181] Asquith M, Powrie F (2010). An innately dangerous balancing act: intestinal homeostasis, inflammation, and colitis-associated cancer. J Exp Med.

[182] Greten FR, Eckmann L, Greten TF, Park JM, Li ZW, Egan LJ, Kagnoff MF, Karin M (2004). IKKbeta links inflammation and tumorigenesis in a mouse model of colitis-associated cancer. Cell.

[183] Nenci A, Becker C, Wullaert A, Gareus R, van Loo G, Danese S, Huth M, Nikolaev A, Neufert C, Madison B, Gumucio D, Neurath MF, Pasparakis M (2007). Epithelial NEMO links innate immunity to chronic intestinal inflammation. Nature.

[184] Lee SH, Hong B, Sharabi A, Huang XF, Chen SY (2009). Embryonic stem cells and mammary luminal progenitors directly sense and respond to microbial products. Stem Cells.

[185] Uematsu S, Jang MH, Chevrier N, Guo Z, Kumagai Y, Yamamoto M, Kato H, Sougawa N, Matsui H, Kuwata H, Hemmi H, Coban C, Kawai T (2006). Detection of pathogenic intestinal bacteria by Toll-like receptor 5 on intestinal CD11c+ lamina propria cells. Nat Immunol.

[186] Integrative HM, and Integrative HMP (iHMP) Research Network Consortium (2014). The Integrative Human Microbiome Project: dynamic analysis of microbiome-host omics profiles during periods of human health and disease. Cell Host Microbe.

[187] D’Abaco GM, Whitehead RH, Burgess AW (1996). Synergy between Apc min and an activated ras mutation is sufficient to induce colon carcinomas. Mol Cell Biol.

[188] Janssen KP, Alberici P, Fsihi H, Gaspar C, Breukel C, Franken P, Rosty C, Abal M, El Marjou F, Smits R, Louvard D, Fodde R, Robine S (2006). APC and oncogenic KRAS are synergistic in enhancing Wnt signaling in intestinal tumor formation and progression. Gastroenterology.

[189] Zhang W, Li F, Nie L (2010). Integrating multiple ‘omics’ analysis for microbial biology: application and methodologies. Microbiology.

[190] Segata N, Boernigen D, Tickle TL, Morgan XC, Garrett WS, Huttenhower C (2013). Computational meta’omics for microbial community studies. Mol Syst Biol.

[191] Borody TJ, Warren EF, Leis S, Surace R, Ashman O (2003). Treatment of ulcerative colitis using fecal bacteriotherapy. J Clin Gastroenterol.

[192] Hedin C, Whelan K, Lindsay JO (2007). Evidence for the use of probiotics and prebiotics in inflammatory bowel disease: a review of clinical trials. Proc Nutr Soc.

[193] Mills S, Shanahan F, Stanton C, Hill C, Coffey A, Ross RP (2013). Movers and shakers: influence of bacteriophages in shaping the mammalian gut microbiota. Gut Microbes.

[194] Howcroft TK, Campisi J, Louis GB, Smith MT, Wise B, Wyss-Coray T, Augustine AD, McElhaney JE, Kohanski R, Sierra F (2013). The role of inflammation in age-related disease. Aging (Albany NY).

